# Identification, Comparison, and Validation of Robust Rumen Microbial Biomarkers for Methane Emissions Using Diverse *Bos Taurus* Breeds and Basal Diets

**DOI:** 10.3389/fmicb.2017.02642

**Published:** 2018-01-09

**Authors:** Marc D. Auffret, Robert Stewart, Richard J. Dewhurst, Carol-Anne Duthie, John A. Rooke, Robert J. Wallace, Tom C. Freeman, Timothy J. Snelling, Mick Watson, Rainer Roehe

**Affiliations:** ^1^Scotland's Rural College, Future Farming System (FFS), Edinburgh, United Kingdom; ^2^Edinburgh Genomics, The Roslin Institute and R(D)SVS, University of Edinburgh, Edinburgh, United Kingdom; ^3^Rowett Institute of Nutrition and Health, University of Aberdeen, Aberdeen, United Kingdom; ^4^Division of Genetics and Genomics, The Roslin Institute and R(D)SVS, University of Edinburgh, Edinburgh, United Kingdom

**Keywords:** rumen microbiome, methane, biomarkers, metagenomics, diets

## Abstract

Previous shotgun metagenomic analyses of ruminal digesta identified some microbial information that might be useful as biomarkers to select cattle that emit less methane (CH_4_), which is a potent greenhouse gas. It is known that methane production (g/kgDMI) and to an extent the microbial community is heritable and therefore biomarkers can offer a method of selecting cattle for low methane emitting phenotypes. In this study a wider range of *Bos Taurus* cattle, varying in breed and diet, was investigated to determine microbial communities and genetic markers associated with high/low CH_4_ emissions. Digesta samples were taken from 50 beef cattle, comprising four cattle breeds, receiving two basal diets containing different proportions of concentrate and also including feed additives (nitrate or lipid), that may influence methane emissions. A combination of partial least square analysis and network analysis enabled the identification of the most significant and robust biomarkers of CH_4_ emissions (VIP > 0.8) across diets and breeds when comparing all potential biomarkers together. Genes associated with the hydrogenotrophic methanogenesis pathway converting carbon dioxide to methane, provided the dominant biomarkers of CH_4_ emissions and methanogens were the microbial populations most closely correlated with CH_4_ emissions and identified by metagenomics. Moreover, these genes grouped together as confirmed by network analysis for each independent experiment and when combined. Finally, the genes involved in the methane synthesis pathway explained a higher proportion of variation in CH_4_ emissions by PLS analysis compared to phylogenetic parameters or functional genes. These results confirmed the reproducibility of the analysis and the advantage to use these genes as robust biomarkers of CH_4_ emissions. Volatile fatty acid concentrations and ratios were significantly correlated with CH_4_, but these factors were not identified as robust enough for predictive purposes. Moreover, the methanotrophic *Methylomonas* genus was found to be negatively correlated with CH_4_. Finally, this study confirmed the importance of using robust and applicable biomarkers from the microbiome as a proxy of CH_4_ emissions across diverse production systems and environments.

## Introduction

Recent metagenomic analyses have highlighted the exciting opportunity that rumen microbial biomarkers of methane (CH_4_) emissions could enable the selection by breeding of cattle which emit less CH_4_ and ultimately may lower agricultural greenhouse gas (GHG) emissions. Ross et al. (2013) highlighted that this approach may surpass current prediction accuracies that are based on the host genome, especially for traits that are difficult to measure and largely influenced by the gut microbiome. Methane has a large impact on global warming, being 28-fold more potent as a GHG than carbon dioxide (CO_2_) (IPCC, 2014). It is one of the main anthropogenic sources (IPCC, 2014) and ruminants are major producers of CH_4_, accounting for 37% of total GHG from agriculture in the UK (Cottle et al., 2011). Methane results as an end product of anaerobic microbial fermentation in the rumen and it significant negative economic and environmental impacts on animal production (Johnson and Johnson, 1995). A limited number of archaeal taxa within Euryarchaeota are methane producers and the genes involved in this process are well-characterized (Thauer et al., 2008; Leahy et al., 2010; Borrel et al., 2013). The hydrogenotrophic pathway catalyzing the conversion of CO_2_ to methane is dominant in the rumen, and occurs in *Methanobrevibacter* spp. (Hook et al., 2010; Danielsson et al., 2017). However, methylotrophic methanogenesis also occurs in the *Methanomassilliicoccales* group (Li et al., 2016), converting methylamine or methanol derived from digestion of feed constituents to methane (Poulsen et al., 2013; Vanwonterghem et al., 2017). In addition, more work is needed to identify the bacterial populations interacting with methanogens for H_2_ or involved in different metabolic pathways associated with lactate or volatile fatty acids (VFA) including propionate, butyrate, or acetate which are known to impact differently methane emissions (Moss et al., 2000; Janssen, 2010; Wanapat et al., 2015; Kamke et al., 2016). For example, *Megasphaera elsdenii* is the major rumen bacterium involved in the acrylate pathway converting lactate to propionate and, in the absence of lactate, producing acetate and butyrate but not propionate from glucose (Hino et al., 1994; Russell and Wallace, 1997). Higher abundance of bacteria populations involved in propionate metabolism is associated with reduced methane emissions compared to acetate metabolism because more H_2_ is utilized per mole VFA thus reducing availability for methane production (Janssen, 2010; Wanapat et al., 2015). Methanotrophic populations within both archaea and bacteria are known to metabolize methane as a carbon and energy source but the impact of such populations in the rumen seems likely to be minor (Parmar et al., 2015; Wallace et al., 2015).

Strategies to lower methane emissions in animal production are becoming an important field of research with the aims to enhance fermentation end-products that are useful to the host and reduce GHG emissions (Immig et al., 1996; Knapp et al., 2014). It is well-known that diet has an impact on the microbial community composition and the genes carried by these populations (Rooke et al., 2014; Henderson et al., 2015). Diets with a higher content of concentrate (e.g., grain) compared to a forage diet (e.g., grass and silages) tend to produce lower methane emissions. For example, Giger-Reverdin and Sauvant (2000) observed that maximum methane emissions occurred between 30 and 40% of grain-based concentrate in the diet. Many feed additives have been explored for their impact on methane emissions. Addition of nitrate or polyunsaturated lipids (e.g., from rapeseed or linseed oil) to the diet showed promising results (Veneman et al., 2015; Guyader et al., 2016). The percentage of concentrate as constituent of the diet strongly affected this inhibitory effect (Duthie et al., 2017). Mechanisms behind this effect are partly explained by the possible inhibition of H_2_ producers in the presence of oil whilst nitrate is thought to act as a competitor with methanogens for H_2_ and may also be toxic to methanogens (Guyader et al., 2015). Besides the use of different diets or additives, recent research has identified links between the rumen microbiome and the host animal (Roehe et al., 2016; Duthie et al., 2017; Malmuthuge and Guan, 2017) and it has been established that host genetics influences methane emissions (Pinares-Patiño et al., 2013; Herd et al., 2014). The rumen microbiome may be the link between host genetics and methane emissions. Therefore, the impact of basal diets, additives and breeds on the microbiome may be considered and evaluated for the identification of robust biomarkers of CH_4_ emissions.

Until now, proxies to predict methane emission phenotypes based on rumen samples including phylogenetic, genomic, or metabolomic markers have not been considered to be robust and accurate, and are also expensive (Negussie et al., 2017). This limitation has been partly attributed to the low number of ruminants studied for the identification and validation of biomarkers. There are inherent difficulties comparing the results of direct quantitation of methanogens using qPCR across different studies due to differences in sampling methods or primer target (McCartney et al., 2013). Quantitative PCR has produced conflicting results when correlating absolute methanogen abundance with CH_4_ emissions (Mosoni et al., 2011; Morgavi et al., 2012). However, a stronger correlation was obtained calculating relative abundance between the Archaea and Bacteria abundance (A:B ratio) in rumen digesta samples (Wallace et al., 2014).

Reliable knowledge about the relationship between CH_4_ emissions and both the microbiome and the metabolites released is very important for improving the identification of biomarkers (McCartney et al., 2013; Ross et al., 2013).

Metagenomics permits the identification of all genes comprising the microbiome and enables taxonomic characterization of the microbial population. Metagenomics has been confirmed to be a powerful method for studying the rumen microbiome (Roehe et al., 2016; Wallace et al., 2017). Roehe et al. (2016) identified 20 genes as biomarkers of methane emissions using a combination of metagenomics and partial least square analyses. Moreover, the same authors showed that these genes clustered together within a genetic network providing a proof of principle about the feasibility of breeding selection by targeting these genes within the rumen microbiome. These preliminary results were obtained on a limited number of beef cattle (*n* = 8) selected as extreme methane emitters (low or high) and fed with two basal diets (forage or concentrate). Therefore, the possibility to use a large scale method like metagenomics on a set of data from different breeds of beef cattle fed different diets and coupled with VFA monitoring is a great opportunity to identify and validate robust biomarkers of CH_4_ emissions.

The aim of this study was (i) to evaluate the effect of two basal diets, and additives on the rumen microbiome of a selection of four beef livestock breeds and to identify robust biomarkers of CH_4_ emissions associated with the microbiome or microbial activities, (ii) the identification of robust biomarkers of CH_4_ emissions associated with data from the microbial community composition, the relative abundance of microbial populations, the relative abundance of genes within the microbiome or VFA concentrations, all data collected from three independent experiments, and (iii) the comparison of these biomarkers to identify those highly correlated with CH_4_ emissions across diverse breeds and diets and the evaluation of the possibility of implementing a breeding strategy using these microbial biomarkers from the rumen microbiome.

## Materials and methods

### Ethics statement

This study was conducted at the Beef and Sheep Research Centre of Scotland's Rural College (SRUC, Edinburgh, UK). The experiment was approved by the Animal Experiment Committee of SRUC and was conducted in accordance with the requirements of the UK Animals (Scientific Procedures) Act 1986.

### Animals, experimental design, and diets

**Figure 1:**
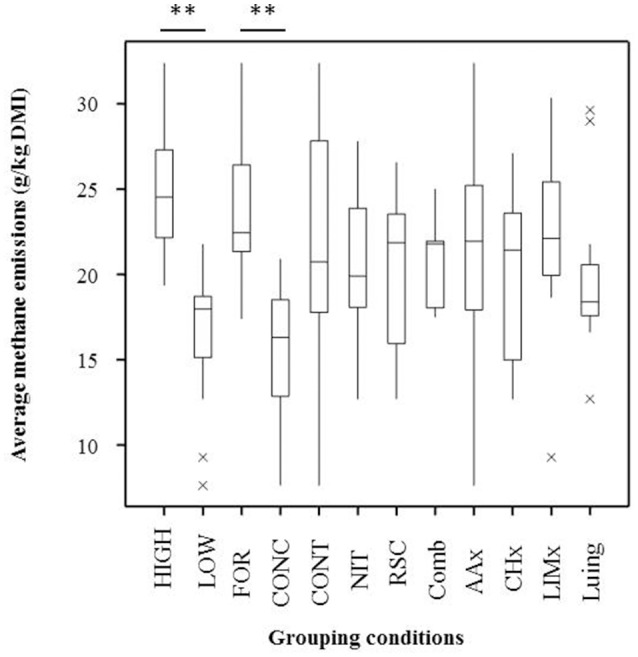
Boxplots representing methane emissions under different conditions. High, High methane emitters (*n* = 25); Low, Low methane emitters (*n* = 25); FOR, Forage (*n* = 34); CONC, Concentrate (*n* = 16); CONT, all controls (*n* = 20); NIT, all samples with nitrate (*n* = 12); RSC, all samples with supplementary lipid (*n* = 12); Comb, all samples with nitrate and supplementary lipid (*n* = 6); AAx, all samples from Aberdeen Angus (*n* = 13); CHx, all samples from Charolais (*n* = 12); LIMx, all samples from Limousin (*n* = 13); Luing: all samples from Luing (*n* = 12). ^**^P < 0.01.

In our previous study (Wallace et al., 2015; Roehe et al., 2016), data on feed efficiency and methane emissions (measured using respiration chambers) were obtained from a 2 × 2 factorial design experiment of breed types and diets using 72 steers from a two-breed rotational cross between Aberdeen Angus (AA) and Limousin (LIM) and completed in 2011. Similar experiments were carried out using purebred Luing (LU) and crossbred Charolais (CH) steers in 2013 and Aberdeen Angus (AA) and Limousin (LIM) rotational crossbred steers in 2014. The data in this study were obtained from samples from those experiments whereby animals with extreme high and low methane emissions (2011) or feed conversion efficiency (2013 and 2014) were selected for whole genome sequencing. The breed type were balanced within experiment comprising 4 AA and 4 LIM in 2011, 9 LU and 9 CH in 2013, and 12 AA and 12 LIM in 2014. Methane emissions were measured individually for 48 h in respiration chambers (Rooke et al., 2014) and based on this result, 25 animals were considered as low CH_4_ emitters whilst the other 25 animals were classified as high CH_4_ emitters. The average CH_4_ emissions (g/kg DMI) between Low and High CH_4_ emitters were significantly different as shown in Figure 1. The animals were offered two complete diets *ad libitum* consisting (g/kg DM) of ~500 forage to 500 concentrate or 80 forage to 920 concentrate which are subsequently referred to as forage and concentrate diets, respectively. Nitrate, lipids, or the combination of both were also added to the basal diet and were compared with the control fed with the same diet without additive. The detailed diet composition and proximate analysis has been reported previously by Rooke et al. (2014) and Duthie et al. (2016, 2017). Animals were fed *ad libitum* during the entire experiment including in the respiration chamber. A single sample of rumen fluid for VFA analysis (expressed as molar proportions) was taken by stomach tube (naso ruminal sampling) within 1 h of cattle leaving the chambers in the 2011 experiment. VFA were determined in 2013 and 2014 using samples collected directly at the abattoir. As recommended by Terré et al. (2013), we compared the VFA profiles between samples rather than total VFA concentrations because of the different methods for rumen sampling applied. The acetate-to-propionate ratio was calculated and considered as a proxy for H_2_ generation.

Samples were obtained from a total of 50 animals balanced for breed type and diet and including the eight post mortem samples previously studied in Roehe et al. (2016), (Table S1).

### Genomic analysis

The animals were fed *ad libitum* until they left the farm and thereafter slaughtered within 2 h in a commercial abattoir where two rumen fluid samples (~50 mL) were taken immediately after the rumen was opened to be drained. The main advantage to collect rumen contents after slaughter is to obtain samples representative of both solid and liquid phases. DNA was extracted from the rumen digesta samples following the protocol described in Rooke et al. (2014).

Illumina TruSeq libraries were prepared from genomic DNA and sequenced on an Illumina HiSeq 2500 instrument (2011 samples) and on an Illumina HiSeq 4000 instrument (2013 and 2014 samples) by Edinburgh Genomics (Edinburgh, UK). Bioinformatics analyses using the two sets of data followed the same procedure as previously described in Wallace et al. (2015). Briefly, functional genes including the genes detailed in this study were identified using KEGG genes database (http://www.kegg.jp). Genes with a relative abundance greater than 0.001% were carried forward for downstream analysis.

For 16S rRNA gene analysis, the genomic reads were aligned to the Greengenes database (Desantis et al., 2006) using Novoalign (www.novocraft.com) and also using the Kraken database (Wood and Salzberg, 2014).

Parameters were adjusted such that all hits were reported that were equal in quality to the best hit for each read, and allowing up to a 10% mismatch across the fragment. Further details are included in Wallace et al. (2015). These data can be downloaded from the European Nucleotide Archive under accession PRJEB10338 and PRJEB21624.

### Statistical analysis

Statistical analysis of the metagenomics samples was based on the complete sample profiles as expressed by the pattern of metagenomic reads classified within KEGG ortholog groups with >90% similarity and belonging to a single KEGG ortholog (KO) groups and the relative abundance (percentage) of individual KO group in each profile. Principal coordinate analysis (PCoA) was carried out using Gen-Stat 16th edition (VSN International Ltd, UK) to identify the factors explaining differences observed in the microbial community (phylum level) between samples. Relative abundance of microbial populations and functional genes, Archaea-to-Bacteria (A:B) ratio, Firmicutes-to-Bacteroidetes (F:B) ratio as an indicator of degradation activities carried by the two main phyla in rumen and acetate-to-propionate ratio were compared using General Linear Models and *P*-values were Bonferroni corrected for multiple testing (SPSS Statistics 22, IBM, USA).

In a network analysis using BioLayout Express3D (Freeman et al., 2007), we identified the distinct functional clusters of microbial genes for each experiment. These networks consist of nodes representing microbial genes and the connecting edges determining the functional linkages between these genes.

Partial least squares analysis (PLS, Version 9.1 for Windows, SAS Institute Inc., Cary, NC, USA) was used to identify the most correlated microbial populations (at the phylum or genus level) or microbial genes associated with methane emissions. This method was successfully applied for the identification of microbial biomarkers in Wallace et al. (2015) and Roehe et al. (2016). The PLS analysis accounted for multiple testing and the correlation between microbial populations or genes as microbial parameters. In addition to microbial parameters, the model included the diet effect (abiotic effector) and additionally the breed type effect (host genetics effect). The model selection was based on the variable importance for projection (VIP) criterion (Wold, 1995), whereby microbial parameters with a VIP <0.8 contribute little to the prediction. Finally, a comparison between different factors identified as highly correlated with CH_4_ emissions and therefore considered as potential biomarkers were tested by PLS analysis. In this study, biomarkers of CH_4_ emissions will be considered as robust when a similar result is observed across diverse diets and breeds and by comparing all potential biomarker together. A robust biomarker may strengthen the confidence of identifying low- vs. high-emitting cattle. Those factors identified to be significant from the microbial community composition, the relative abundance of microbial populations or genes or VFA concentrations. All samples without VFA measurements were removed (N1, N3, N7, and RR41).

The residual methane emissions were calculated using a General Linear Model including diet and breed into the model and measured methane data as dependent variable. These residual methane emissions are thus corrected for diet and breed and were centered and standardized and only used when biomarkers were compared together.

Spearman's correlation analysis was also carried out to determine which factors (the same factors tested by PLS) are correlated with CH_4_ emissions using SPSS Statistics 22. *P*-values ≤ 0.05 were considered significant and tendencies were represented (*P*-values < 0.1).

## Results

### Factors influencing the differences observed in methane emissions

Several grouping conditions were tested using methane emission values from three independent trials (Figure 1). Average CH_4_ emissions were 20.89 ± 0.75 g/kg dry matter intake based on measurements from 50 animals. CH_4_ emissions were 1.48-fold higher in the high-CH_4_ group (*P* < 0.001). CH_4_ emissions were also higher in animals fed the forage compared to concentrate basal diet (*P* < 0.001).

CH_4_ emissions showed strong correlations with acetate (*F* = 0.582, *P* < 0.001), propionate (*F* = −0.574, *P* < 0.001), and valerate (*F* = −0.571, *P* < 0.001) concentrations and to a lesser extent isovalerate concentration (*F* = −0.347, *P* < 0.05) but not with butyrate or isobutyrate concentrations (Table S1). Acetate-to-propionate ratio was strongly positively correlated (*P* < 0.001) with CH_4_ emissions (Figure S1A). When samples were divided based on diet treatment, this significant correlation disappeared in presence of concentrate (Figure S1B) and only a tendency was found with the forage diet (*P* = 0.08; Figure S1C).

### Change in microbial community composition between CH_4_ emitters and diet treatments

Using the Kraken database for the identification of the 16S rRNA sequences (Phylum level) within the 50 metagenomics datasets, the difference observed within the microbial community composition represented 36.9% over the first two principal coordinate analysis axes (Figure S2) and 45.2% when the third axis was included (data not shown).

The most abundant bacterial phyla (on average) identified were Firmicutes (42.8%), Bacteroidetes (38.6%), Proteobacteria (6.6%), Fibrobacteres (4.9%), and Actinobacteria (2.4%) representing on average 95.3% of the total community (Figure S3). Proteobacteria was the only dominant phylum significantly different with a higher abundance in low-CH_4_ samples compared to high-CH_4_ samples (*P* = 0.03). Lower abundant phyla such as Deinococcus-Thermus (0.12%, *P* = 0.006), Chlorobi (0.07%, *P* = 0.003), Kiritimatiellaeota (0.01%, *P* = 0.02), Verrucomicrobia (0.12%, *P* = 0.04), and Calditrichaeota (0.003%, *P* = 0.01) were also identified as significantly different and generally with a higher abundance in high-CH_4_ samples except for Calditrichaeota. Comparing the effect of forage or concentrate diets, a limited number of bacterial phyla (*n* = 4/32) were affected, which were based on their relative abundance in the rumen minor populations (Table S2). In general, the relative abundance of microbial populations impacted by additives was higher in control treatment except Calditrichaeota and Proteobacteria, the latter being 1.2-fold higher in presence of nitrate compared to the control concentrate treatment (Table S2). On average, the Firmicutes-to-Bacteroidetes ratio was at 1.22 and not significantly different between methane emitters or diet treatments. Euryarchaeota were not impacted by nitrate or RSC in either concentrate or forage diets.

**Figure 2:**
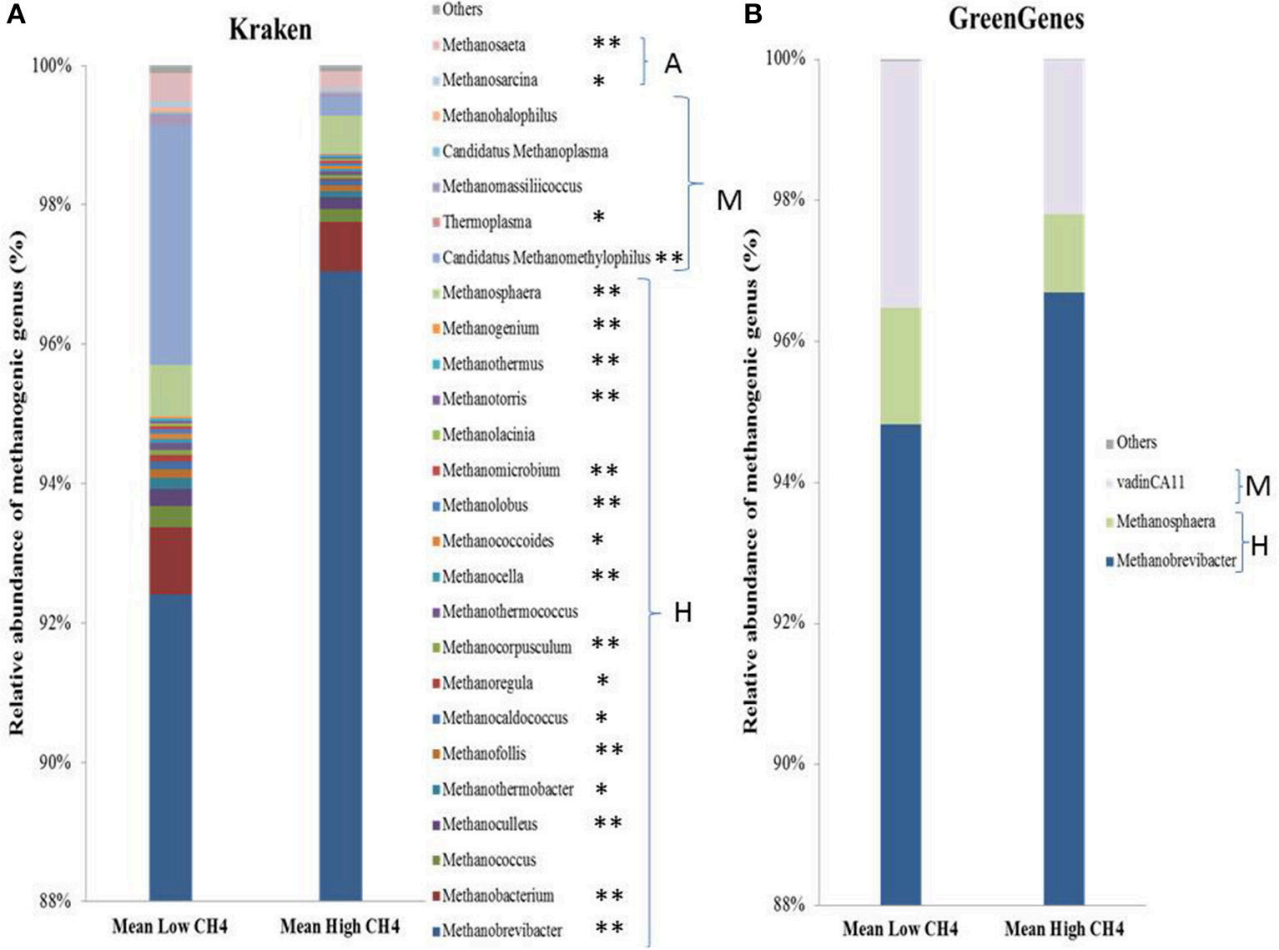
Diversity of methanogen genera using **(A)** Kraken database or **(B)** Greengenes database. A, Acetoclastic methanogens; H, Hydrogenotrophic methanogens; M, Methylotrophic methanogens. ^**^P < 0.01, ^*^P < 0.05 indicates different between low and high emitting groups.

The archaeal community represented 5.33 ± 0.37% of the total microbial community based on 16S rRNA sequences and higher Shannon diversity was characterized using the Kraken database compared to Greengenes as shown in Figure 2A, with the former identifying more methanogenic groups capable of utilizing acetoclastic, hydrogenotrophic, and methylotrophic pathways to produce methane (Figure S4). The hydrogenotrophic pathway was highly represented in the rumen content of both high- and low-methane emitting animals, mostly in high emitters and represented on average 96.8% of total methanogens (Figure S4A). The relative abundance of total methanogens was double in high emitters compared to low emitters. This result was explained by the significant dominance of several populations including *Methanobrevibacter* (on average 94% of the methanogens)*, Methanobacterium, Methanococcus*, and *Methanoculleus* species (Figure 2A). On the other hand, the dominant methylotrophic methanogen belonging to *Methanomassiliicoccales* order was identified as Candidatus *Methanomethylophilus*, with a relative abundance 7-fold significantly higher in the rumen microbiome of low-methane emitters compared to high-methane rumen samples (Figure 2A). Finally, the dominant acetoclastic methanogen was *Methanosarcina* species and represented on average 0.4% of total methanogens (Figure 2A). Overall, Shannon diversity index calculated for total microbial community did not show any significant differences between groups of methane emitters or diet. Focusing on methanogens, a higher diversity in low emitters was confirmed with a Shannon diversity index of 0.55 compared to 0.28 in high emitters (*P* < 0.001). Effect of the additives on the relative abundance of methanogen populations was not significant whilst methylotrophic methanogenic populations were on average 2.27-fold more abundant in the concentrate diet supplemented with nitrate compared to the control condition and only on average 1.21-fold higher in forage diet supplemented with nitrate compared to the control treatment.

Using the Greengenes annotation, both methanogen diversity (Shannon index H) and composition were lower and only represented by three dominant genera. However, the general results on the dominant populations, methanogen diversity and the importance of methylotrophic methanogens in low-methane emitters were the same but it has to be considered that using this database the minor populations (e.g., acetoclastic methanogens) were not recovered (Figure 2B and Figure S4B).

Methanotrophic populations were also identified when using the Kraken database, representing a limited part of the microbial community and being about 70-fold less abundant than methanogens (on average 0.1 ± 0.01%). This microbial group was highly dominated by three methanotrophic bacteria including the genus *Methylobacterium* and to a lesser extent *Methylomonas* and *Methylomicrobium* genera. However, only the *Methylomonas* genus was different between emitters (*P* = 0.005) or diet treatments (*P* = 0.005) with a relative abundance 1.7-fold higher in low- compared to high-methane emitters. Finally, the diversity of methanotrophic organisms was greater in high emitters (*P* = 0.02) compared to low emitters and there was no effect of diet or additives on methanotrophic populations.

### Identification of additional phylogenetic biomarkers of methane emissions

**Figure 3:**
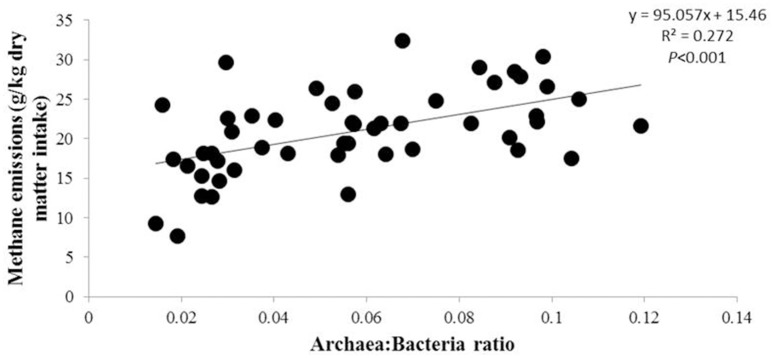
Linear regression between Archaea:Bacteria ratio and CH_4_ emissions. Black circle: all samples. Equation for the linear regression was included in figure when the difference was significant (*P* < 0.05).

The Archaea:Bacteria ratio was calculated for each sample and a positive correlation (*P* < 0.001) was confirmed by linear regression with methane emissions overall (Figure 3). This correlation was weaker when samples were grouped based on diet—being significant (*P* < 0.01) for the concentrate but not the forage diet (Figure S5). Interestingly, a positive correlation between CH_4_ emissions and the relative abundance of Euryarchaeota was confirmed (*F* = 0.567, *P* = 0.003) but only when studying high emitters.

Partial Least Square analysis including in the model diet and breed effects showed that the relative abundances of 31 microbial genera were negatively correlated with methane emissions (“Reducing effects on methane emissions” group in Table 1). There were 56 genera positively correlated (including 16 highly positively correlated) with methane (“Increasing effects on methane emissions” group in Table 1) and 40 genera considered as positively correlated with methane emissions but showing a low regression coefficients (“Low effect on methane emissions” in Table 1). Moreover, the result generated by PLS and including the 56 genera, breed type and diet effects, explained 50% of the variation in CH_4_ emissions. One main result is that bacterial populations showed higher VIP value compared to methanogens including the most abundant genus *Methanobrevibacter* and four other hydrogenotrophic methanogens present at lower abundance including *Methanosphaera* genus. Bacteria producing butyrate (e.g., *Butyrivibrio* and *Pseudobutyrivibrio* spp.) or CO_2_ were positively correlated with CH_4_ emissions, contrasting with those associated with amino acid (e.g., *Acidaminococcus* and *Allisonella* species) and lactate metabolism (e.g., *Megasphaera* and *Lactobacillus* genera) or populations consuming hydrogen (e.g., *Dehalococcoides* genus). Other bacterial populations with significant VIP were known to be associated with nitrogen (*Nitrosococcus* or *Nitrobacter* spp.) or sulfur cycles or those classified in the average group were halotolerant populations or potentially involved in organic matter breakdown, or syntrophic activities (e.g., *Syntrophobotulus* genus).

**Table 1:** PLS results identifying the most important microbial genera affecting methane emissions.

**Phylum**	**Microbial Genus**	**VIP**	**Coef**.	**Mean Low CH4**	**Mean High CH4**	**L/H CH4 ratio**	**Function**
**REDUCING EFFECTS ON METHANE EMISSIONS**							
Chloroflexi	*Dehalococcoides*	1.42	−0.039	0.044	0.020	2.15	H_2_ ox.
Bacteroidetes	*Odoribacter*	1.39	−0.040	0.058	0.028	2.09	Commensal
Firmicutes	*Megasphaera*	1.35	−0.037	0.218	0.047	4.68	Lactate
Firmicutes	*Acidaminococcus*	1.34	−0.035	1.143	0.099	11.57	AA
Firmicutes	*Jeotgalicoccus*	1.23	−0.035	0.003	0.002	1.17	Halotolerant
Firmicutes	*Allisonella*	1.16	−0.026	0.057	0.005	11.17	AA
Firmicutes	*Salinicoccus*	1.11	−0.028	0.004	0.003	1.57	Halotolerant
Thermotogae	*Kosmotoga*	1.09	−0.028	0.003	0.002	1.31	Thermophile
Bacteroidetes	*Mitsuokella*	1.08	−0.022	0.533	0.070	7.59	Phytate
Actinobacteria	*Olsenella*	1.07	−0.029	2.151	0.983	2.19	Lactate
Bacteroidetes	*Bacteroides*	1.02	−0.027	1.697	1.102	1.54	VFA
Firmicutes	*Dorea*	0.98	−0.024	0.106	0.062	1.70	Acetogen
Proteobacteria	*Wenzhouxiangella*	0.98	−0.001	0.016	0.005	3.28	Halotolerant
Firmicutes	*Roseburia*	0.96	−0.022	0.172	0.079	2.18	Butyrate
Proteobacteria	*Edwardsiella*	0.96	−0.003	0.032	0.019	1.71	N.I.
Firmicutes	*Aneurinibacillus*	0.96	−0.026	0.005	0.003	1.66	Lignin degrader
Firmicutes	*Pelosinus*	0.96	−0.023	0.017	0.011	1.48	Degrader
Proteobacteria	*Methylomonas*	0.95	−0.002	0.018	0.011	1.73	Methanotrophy
Firmicutes	*Veillonella*	0.94	−0.019	0.008	0.003	2.57	Lactate
Proteobacteria	*Halotalea*	0.94	−0.003	0.009	0.005	1.83	Halotolerant
Proteobacteria	*Alkalilimnicola*	0.92	−0.001	0.010	0.007	1.41	Halotolerant
Proteobacteria	*Sulfurovum*	0.92	−0.017	0.007	0.004	1.46	H_2_ ox.
Proteobacteria	*Colwellia*	0.92	−0.016	0.007	0.003	2.25	Alkane degrader
Proteobacteria	*Marinomonas*	0.91	−0.016	0.004	0.003	1.39	Halotolerant
Proteobacteria	*Nitrobacter*	0.90	0.000	0.009	0.006	1.42	NOB
Proteobacteria	*Thalassospira*	0.90	−0.023	0.003	0.003	1.16	Halotolerant
Firmicutes	*Faecalitalea*	0.87	−0.024	0.020	0.010	2.02	AA
Euryarchaeota	*Methanohalophilus*	0.85	−0.022	0.002	0.001	1.50	Methanogen (M)
Firmicutes	*Lactobacillus*	0.83	−0.019	0.338	0.199	1.70	Lactate
Bacteroidetes	*Zobellia*	0.83	−0.021	0.003	0.002	1.27	Mesophile
Proteobacteria	*Nitrosococcus*	0.83	−0.022	0.003	0.002	1.26	AOB
**LOW EFFECT ON METHANE EMISSIONS**							
Actinobacteria	*Sanguibacter*	1.11	0.012	0.011	0.006	1.72	In blood
Proteobacteria	*Aromatoleum*	1.06	0.016	0.008	0.007	1.26	Degrader
Proteobacteria	*Thiocystis*	1.04	0.009	0.014	0.010	1.46	Sulfur
Proteobacteria	*Microbulbifer*	1.02	0.005	0.022	0.010	2.17	Halotolerant
Euryarchaeota	*Halosimplex*	1.02	0.010	0.004	0.002	1.81	Halotolerant
Proteobacteria	*Cronobacter*	1.00	0.002	0.042	0.020	2.08	Pathogen
Actinobacteria	*Modestobacter*	0.99	0.004	0.010	0.005	2.19	Halotolerant
Proteobacteria	*Neorickettsia*	0.99	0.011	0.002	0.001	1.35	Pathogen
Proteobacteria	*Halorhodospira*	0.98	0.004	0.014	0.008	1.77	Halotolerant
Proteobacteria	*Serratia*	0.98	0.008	0.061	0.048	1.27	N.I.
Spirochaete	*Salinispira*	0.98	0.010	0.008	0.005	1.39	Halotolerant
Proteobacteria	*Asticcacaulis*	0.98	0.007	0.008	0.005	1.59	N.I.
Proteobacteria	*Sideroxydans*	0.98	0.003	0.012	0.005	2.29	Iron ox.
Proteobacteria	*Pantoea*	0.97	0.001	0.043	0.023	1.83	N.I.
Proteobacteria	*Agrobacterium*	0.97	0.007	0.041	0.032	1.29	N.I.
Proteobacteria	*Raoultella*	0.97	0.009	0.012	0.009	1.40	Pathogen
Proteobacteria	*Halomonas*	0.96	0.002	0.043	0.027	1.59	Halotolerant
Armatimonadetes	*Chthonomonas*	0.96	0.002	0.004	0.002	1.82	N.I.
Proteobacteria	*Ferrimonas*	0.96	0.005	0.010	0.006	1.59	Iron
Proteobacteria	*Acidihalobacter*	0.96	0.011	0.017	0.013	1.26	Halotolerant
Actinobacteria	*Dermabacter*	0.95	0.003	0.007	0.004	1.95	N.I.
Proteobacteria	*Dokdonella*	0.95	0.001	0.011	0.007	1.64	N.I.
Proteobacteria	*Enterobacter*	0.95	0.004	0.061	0.046	1.32	Degrader
Actinobacteria	*Tsukamurella*	0.95	0.002	0.007	0.004	1.90	Degrader
Proteobacteria	*Immundisolibacter*	0.95	0.001	0.016	0.009	1.89	Degrader
Proteobacteria	*Mesorhizobium*	0.95	0.007	0.055	0.042	1.31	Degrader
Proteobacteria	*Lacimicrobium*	0.94	0.005	0.004	0.003	1.51	halotolerant
Proteobacteria	*Castellaniella*	0.94	0.008	0.014	0.010	1.33	N.I.
Proteobacteria	*Pseudomonas*	0.94	0.001	0.482	0.342	1.41	Degrader
Proteobacteria	*Defluviimonas*	0.94	0.003	0.008	0.005	1.49	Halotolerant
Dienococcus-Thermus	*Truepera*	0.93	0.007	0.009	0.007	1.28	Degrader
Proteobacteria	*Methyloceanibacter*	0.93	0.003	0.008	0.006	1.40	Methylotrophy
Proteobacteria	*Thioflavicoccus*	0.93	0.004	0.015	0.011	1.40	Sulfur
Firmicutes	*Syntrophobotulus*	0.92	0.003	0.008	0.005	1.49	Syntrophy
Proteobacteria	*Dyella*	0.91	0.004	0.028	0.021	1.33	Degrader
Chlorobi	*Chlorobium*	0.90	0.002	0.021	0.016	1.32	Sulfur
Cyanobacteria	*Microcoleus*	0.90	0.004	0.002	0.002	1.33	Sulfur
Proteobacteria	*Chelativorans*	0.89	0.006	0.010	0.007	1.29	Degrader
Proteobacteria	*Halioglobus*	0.89	0.002	0.005	0.004	1.36	Halotolerant
Proteobacteria	*Pluralibacter*	0.88	0.003	0.014	0.010	1.33	Pathogen
**INCREASING EFFECTS ON METHANE EMISSIONS**							
Proteobacteria	*Sedimenticola*	1.36	0.038	0.008	0.005	1.40	SOB
Firmicutes	*Sarcina*	1.33	0.038	1.142	3.246	0.35	CO_2_ prod.
Firmicutes	*Butyrivibrio*	1.31	0.037	2.107	3.017	0.70	Butyrate
Euryarchaeota	*Methanotorris*	1.30	0.036	0.002	0.003	0.58	Methanogen (H)
Euryarchaeota	*Methanobrevibacter*	1.23	0.034	4.166	7.146	0.58	Methanogen (H)
Planctomycetes	*Isosphaera*	1.15	0.032	0.003	0.005	0.63	Degrader
Firmicutes	*Pseudobutyrivibrio*	1.13	0.032	0.434	0.617	0.70	Butyrate
Euryarchaeota	*Methanobacterium*	1.11	0.032	0.040	0.053	0.76	Methanogen (H)
Planctomycetes	*Singulisphaera*	1.09	0.030	0.005	0.007	0.61	Degrader
Bacteroidetes	*Emticicia*	1.06	0.029	0.003	0.005	0.69	Fucosidase
Verrucomicrobia	*Opitutus*	1.06	0.029	0.012	0.019	0.66	H_2_ producer
Planctomycetes	*Rubinisphaera*	0.99	0.027	0.004	0.006	0.74	CO_2_ prod.
Elusimicrobia	*Endomicrobium*	0.91	0.024	0.005	0.008	0.63	VFA
Euryarchaeota	*Methanocaldococcus*	0.90	0.023	0.004	0.006	0.67	Methanogen (H)
Euryarchaeota	*Methanococcus*	0.86	0.023	0.011	0.015	0.74	Methanogen (H)
Euryarchaeota	*Methanosphaera*	0.86	0.023	0.032	0.041	0.77	Methanogen (H)

*VIP, Variable importance for projection; Coef, Coefficient; AA, Amino acids metabolim; AOB, Ammonia-oxidizing bacteria; NOB, Nitrite-oxidizing bacteria; SOB, Sulfur-oxidizing bacteria; ox, Oxidizer; Methanogen (H), Hydrogenotrophic pathway; Methanogen (M), Methylotrophic methanogenic pathway; VFA, Volatile Fatty Acids; N.I., No information*.

### Validation of functional genes as biomarkers of methane emissions

**Figure 4:**
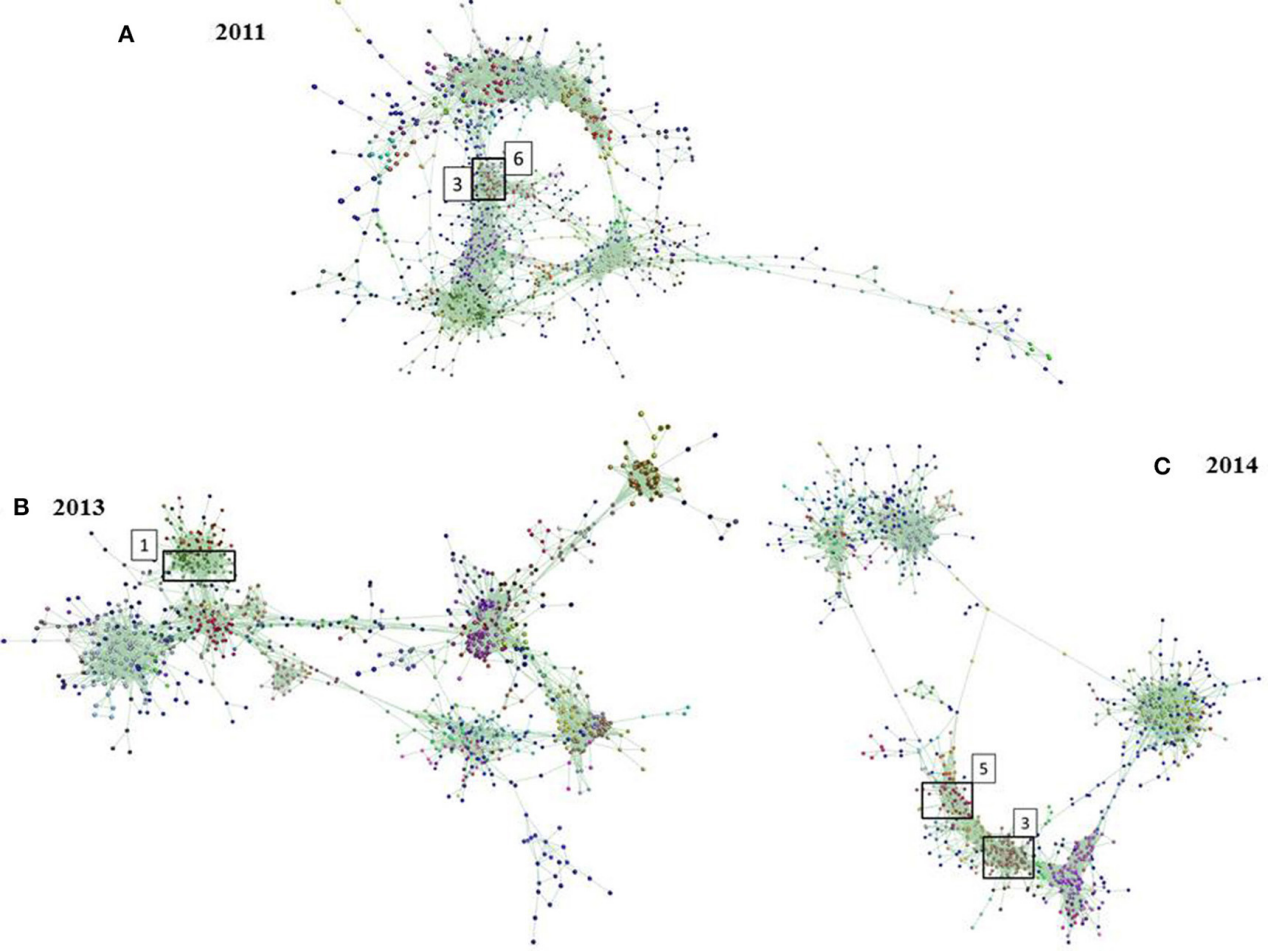
Functional clusters of microbial genes identified using network analysis for **(A)** the 2011 experiment (*n* = 1424 genes), **(B)** the 2013 experiment (*n* = 1178 genes), **(C)** the 2014 experiment (*n* = 1224 genes). Correlation analysis of microbial gene abundance was used to construct networks, where nodes represent microbial genes and edges the correlation in their abundance.

The main result from the network analysis is that most of the same genes directly involved in methane emissions were found over three independent trials and in one or two closed clusters. For example, these genes grouped within a single cluster (C1) for the 2013 samples or two clusters for the 2011 samples (C3 and C6) and 2014 samples (C3 and C5) (Figures 4A–C). Overall, 202 genes representing different microbial functions were identified using KEGG in these clusters including those known to be involved in methane emissions (*n* = 37). However, only 27 genes associated with [high or low] methane emissions were detected in the three experiments.

A PLS analysis using 202 genes (“general analysis”) was carried out and the results are summarized in Table 2. As a result, 37 genes were identified as important to predict methane emissions in cattle and as part of a model including breed type and diet effects explained 62% of the variation in methane emissions. The most abundant of these were either subunits of the methyl coenzyme M reductase gene catalyzing the final step of CH_4_ synthesis pathway *mcrABG* (K00399, K00401 K00402) encoding for or genes associated with hydrogenase activity, such as formate dehydrogenase, tetrahydromethanopterin S-methyltransferase, formylmethanofuran dehydrogenase (K00123, K00125, K00577, K00580, K00581, and K00584) or energy synthesis (V-type H+-transporting ATPase) (K02117 and K02118). The former enzymes are associated with the hydrogenotrophic pathway while the genes encoding for heterodisulfide reductase (K03389, K03390) and associated with low emitters, part of the methylotrophic methanogenic pathway. All these genes were significantly higher in high-emitting rumen samples compared to low-emitters (*P* < 0.02). Finally, the genes with a higher VIP were not those encoding for the final reaction leading to CH_4_ emissions but were associated with the transfer of the methyl group (e.g., K06937) or hydrogen (e.g., K02117 and K02118). In parallel, a similar PLS analysis was carried out but only using the genes (*n* = 36) known to be directly involved in the methane emissions pathway (Table S3). As a result, the percentage of variation in methane emissions explained by these genes increased (65%) compared to the general analysis (62%). Moreover, the genes with a higher VIP were not those encoding for methyl-coenzyme M reductase (Table S3) as observed in the general analysis.

**Table 2:** PLS results identifying the most important functional genes affecting methane emissions.

**KEGG ID**	**Function**	**VIP**	**Coef**.	**Mean Low CH4**	**Mean High CH4**	**L/H CH4 ratio**
**INCREASING EFFECTS ON METHANE EMISSIONS**						
K06937	7,8-dihydro-6-hydroxymethylpterin dimethyltransferase	1.26	0.096	0.007	0.018	0.36
K00046	Gluconate 5-dehydrogenase	1.13	0.089	0.067	0.098	0.68
K02117	V-type H+-transporting ATPase subunit A	1.07	0.067	0.135	0.216	0.62
K02118^*^	V-type H+-transporting ATPase subunit B	1.02	0.057	0.120	0.189	0.63
K00584^*^^a^	Tetrahydromethanopterin S-methyltransferase subunit H	1.00	0.053	0.049	0.103	0.48
K00203 ^a^	Formylmethanofuran dehydrogenase subunit D	0.99	0.046	0.017	0.032	0.53
K00200^*^^a^	Formylmethanofuran dehydrogenase subunit A	0.99	0.042	0.066	0.125	0.53
K00150	Glyceraldehyde-3-phosphate dehydrogenase (NAD(P))	0.98	0.042	0.026	0.056	0.47
K01499 ^a^	Methenyltetrahydromethanopterin cyclohydrolase	0.97	0.037	0.040	0.079	0.50
K00169^*^^a^	Pyruvate ferredoxin oxidoreductase, alpha subunit	0.97	0.034	0.032	0.062	0.52
K00580^*^^a^	Tetrahydromethanopterin S-methyltransferase subunit D	0.95	0.031	0.021	0.045	0.47
K00400^*^^a^	Methyl coenzyme M reductase system, component A2	0.95	0.027	0.022	0.047	0.48
K00170^*^^a^	Pyruvate ferredoxin oxidoreductase, beta subunit	0.94	0.032	0.023	0.044	0.52
K13812^*^^a^	Bifunctional enzyme Fae/Hps	0.94	0.029	0.031	0.062	0.50
K14128^*^^a^	F420-non-reducing hydrogenase subunit G	0.93	0.032	0.046	0.078	0.59
K02303	Uroporphyrin-III C-methyltransferase	0.93	0.060	0.004	0.010	0.44
K14120^*^	Energy-converting hydrogenase B subunit K	0.92	0.064	0.005	0.010	0.46
K00123^*^^a^	Formate dehydrogenase, alpha subunit	0.92	0.007	0.126	0.206	0.61
K00201^*^^a^	Formylmethanofuran dehydrogenase subunit B	0.91	0.028	0.091	0.155	0.58
K01959	Pyruvate carboxylase subunit A	0.91	0.014	0.027	0.051	0.53
K00581^*^^a^	Tetrahydromethanopterin S-methyltransferase subunit E	0.90	0.001	0.055	0.094	0.58
K00672 ^a^	Formylmethanofuran–tetrahydromethanopterin N-formyltransferase	0.89	0.003	0.024	0.056	0.43
K00399^*^^a^	Methyl-coenzyme M reductase alpha subunit	0.89	0.003	0.137	0.223	0.61
K01673	Carbonic anhydrase	0.89	0.059	0.007	0.014	0.46
K00205 ^a^	Formylmethanofuran dehydrogenase subunit F	0.86	0.040	0.012	0.025	0.47
**LOW EFFECTS ON METHANE EMISSIONS**						
K03389	Heterodisulfide reductase subunit B	1.13	−0.061	0.047	0.069	0.68
K00440^*^	Coenzyme F420 hydrogenase alpha subunit	1.10	−0.059	0.039	0.059	0.66
K00320	Coenzyme F420-dependent N5,N10-methenyltetrahydromethanopterin reductase	1.03	−0.054	0.076	0.109	0.70
K14123^*^^a^	Energy-converting hydrogenase B subunit N ^1^	1.02	−0.039	0.011	0.021	0.51
K00202^*^	Formylmethanofuran dehydrogenase subunit C ^1^	1.01	−0.028	0.043	0.066	0.65
K14101	Energy-converting hydrogenase A subunit J ^1^	1.00	−0.051	0.007	0.012	0.55
K00125^*^^a^	Formate dehydrogenase, beta subunit ^1^	1.00	−0.027	0.051	0.082	0.62
K00401	Methyl-coenzyme M reductase beta subunit ^1^	1.00	−0.030	0.089	0.135	0.66
K07388	Hydrogenase expression/formation protein	0.95	−0.024	0.019	0.031	0.60
K00577^*^^a^	Tetrahydromethanopterin S-methyltransferase subunit A ^1^	0.94	−0.005	0.029	0.057	0.51
K03390	Heterodisulfide reductase subunit C	0.93	−0.002	0.023	0.041	0.56
K00402	Methyl-coenzyme M reductase gamma subunit ^1^	0.86	−0.004	0.051	0.076	0.67

1 *Potential reasons for unexpected negative coefficients will be addressed in the discussion*.

* *Genes also identified in the network analysis*.

a *Genes previously identified in Roehe et al. (2016) as biomarkers of methane emissions. VIP, Variable Importance in Projection; Coef, Coefficient*.

### Comparison between the different biomarkers tested and correlation with CH_4_ emissions

Potential biomarkers were compared together by PLS analysis to evaluate the factors highly correlated with CH_4_ emissions (Table S4). Residual CH_4_ emissions data were estimated to remove the effect of diets and breeds and to allow the comparison of the potential biomarkers identified by PLS as significantly correlated with CH_4_ emissions. The PLS results identified 37 factors with a VIP value > 0.80 and explaining 42% of the variation in residual CH_4_ (Table 3). Within the 37 factors, 22 individual genes mostly involved in the hydrogenotrophic methanogen pathway were identified. The other parameters identified included methanogen populations (e.g., *Methanobrevibacter, Methanotorris*, and *Methanohalophilus* genera), the Shannon diversity indices for the methanogen community, PCoA scores or six bacterial populations as well as the Archaea-to-Bacteria ratio. Finally, all the other parameters previously tested and including the Acetate-to-Propionate ratio, or the data on the methanotrophs (relative abundance) were not identified as final biomarkers. A different result was obtained when a Spearman correlation test was applied on the same set of data using non-corrected methane values and therefore still considering the effects of diet and breed (Table S5). For example, Acetate:Propionate ratio showed the highest correlation with CH_4_ emissions.

**Table 3:** PLS analysis comparing potential biomarkers correlated with CH_4_ emissions.

**Factor**	**VIP**	**Coefficient**	**Information**
**PHYLOGENETIC FACTOR**^a^			
*Methanotorris*	1.69	0.14	Hydrogenotrophic methanogen
*Methanobrevibacter*	1.37	0.12	Hydrogenotrophic methanogen
*Methanocaldococcus*	1.25	0.11	Hydrogenotrophic methanogen
*Methanohalophilus*	1.09	−0.09	Methylotrophic methanogen
*Faecalitalea*	0.99	−0.09	AA
*Dorea*	0.90	−0.08	Acetogen
*Colwellia*	0.88	0.04	Alkane degrader
*Opitutus*	0.88	−0.03	H_2_ producer
*Singulisphaera*	0.88	−0.02	Degrader
*Isosphaera*	0.85	−0.03	Degrader
**MICROBIAL COMMUNITY FACTOR**			
PCoA-2^b^	1.55	−0.13	
Met Shannon Even^b^	1.33	−0.12	Methanogen evenness
Met Shannon Div^b^	1.32	−0.12	Methanogen diversity
A:B	0.88	−0.01	Archaea:Bacteria ratio
PCoA-1^b^	0.86	−0.05	
**METAGENOMICS FACTOR**^c^			
K00672	1.32	−0.05	Formylmethanofuran-tetrahydromethanopterin N-formyltransferase
K00581	1.08	−0.02	Tetrahydromethanopterin S-methyltransferase subunit E
K00150	1.06	−0.02	Glyceraldehyde-3-phosphate dehydrogenase (NAD(P))
K01959	1.02	−0.02	Pyruvate carboxylase subunit A
K00580	1.00	−0.01	Tetrahydromethanopterin S-methyltransferase subunit D
K01499	0.95	0.00	Methenyltetrahydromethanopterin cyclohydrolase
K00584	0.94	0.00	Tetrahydromethanopterin S-methyltransferase subunit H
K01673	0.93	−0.03	Carbonic anhydrase
K13812	0.93	0.00	Bifunctional enzyme Fae/Hps
K00123	0.92	0.01	Formate dehydrogenase, alpha subunit
K00400	0.89	0.01	Methyl coenzyme M reductase system, component A2
K00402	0.89	−0.07	Methyl-coenzyme M reductase gamma subunit
K00399	0.89	0.00	Methyl-coenzyme M reductase alpha subunit
K02118	0.87	0.01	V-type H+-transporting ATPase subunit B
K00200	0.87	0.02	Formylmethanofuran dehydrogenase subunit A
K00201	0.86	0.01	Formylmethanofuran dehydrogenase subunit B
K14128	0.85	0.03	F420-non-reducing hydrogenase subunit G
K00169	0.84	0.03	Pyruvate ferredoxin oxidoreductase, alpha subunit
K00170	0.84	0.02	Pyruvate ferredoxin oxidoreductase, beta subunit
K02117	0.84	0.02	V-type H+-transporting ATPase subunit A
K00203	0.82	0.04	Formylmethanofuran dehydrogenase subunit D
K06937	0.80	0.03	7,8-dihydro-6-hydroxymethylpterin dimethyltransferase

a *Value based on the relative abundance of the microbial genera identified as significantly correlated by PLS*,

b *Data obtained by calculating the Shannon diversity indices or doing a PCoA on the relative abundance of the microbial phyla*,

c *Value based on the relative abundance of the genes identified as significantly correlated by PLS*.

## Discussion

### Treatment effects on methane emissions

In the present study, the results of three independent trials were compared and combined, and the current analysis confirmed that the constituent of the basal diet was strongly and significantly associated with CH_4_ emissions. The proportion of dietary forage to concentrate content in the diet as previously identified by Roehe et al. (2016) and Rooke et al. (2014). The dietary additives used in this study as a strategy to lower CH_4_ emissions did not show significant results contrasting with previous works identifying nitrate and supplementary lipid as some of the most promising methane mitigation additives in ruminants (Wallace et al., 2014; Olijhoek et al., 2015; Guyader et al., 2016) while variations in response were detected (Yang et al., 2016). Factors that could explain these differences included the use of a reduced number of rumen samples from animals initially selected for low- and high-feed conversion and also the variability in the basal diet composition.

### Identification of functional genes as biomarkers of methane emissions

In this combined analysis, most of the genes previously identified by Wallace et al. (2015) and Roehe et al. (2016) were in general also identified in this study (*n* = 19/20) by PLS analysis over the three independent experiments and confirmed as strong biomarkers of CH_4_ emissions. Most of these genes were involved in the hydrogenotrophic methane synthesis pathway and grouped in one cluster or two attached clusters over the three independent experiments as previously highlighted by Roehe et al. (2016). This study is one of the first confirming the importance of genes encoding for heterodisulfide reductase in the rumen over the genes associated with methylamine compounds or methanol conversion to accomplish the first step of methylotrophic methanogenic pathway (Buan and Metcalf, 2010; Borrel et al., 2013). Although genes encoding for heterodisulfide reductase were confirmed to be significantly correlated with methane in the rumen of low emitters, the result of the biomarker comparison did not identify those genes as robust biomarkers of CH_4_ emissions. This result confirmed the dominance of hydrogenotrophy over methylotrophy in the rumen (Hook et al., 2010; Danielsson et al., 2017) but also highlighted that both pathways are important in explaining methane emissions (Poulsen et al., 2013). Interestingly, these genes associated with high VIP value were in the upper part of the pathway and encode for methyltransferase, hydrogenase, or dehydrogenase activities but not directly the genes (e.g., *mcrA*) encoding for the methyl coenzyme M reductase system the final step in methane production. This result tends to confirm the importance of hydrogen concentration and thermodynamics affecting the microbial communities and therefore VFA production and methane emissions (Wolin et al., 1997; Rooke et al., 2014). Contrasting with Shi et al. (2014), this study confirmed a significant increase in the relative abundance of most of the genes involved in CH_4_ emissions by metagenomics, but in agreement with the same authors, not only *mcrA*, but all genes are important in explaining higher CH_4_ emissions. These results could explain weak correlations previously observed with CH_4_ emissions when targeting directly 16S rRNA gene or *mcrA* (Morgavi et al., 2012; Tapio et al., 2017). There have been estimated unexpected negative associations of microbial gene abundances and methane emissions (Table 2). Several reasons including bioinformatics limitation (e.g., gene annotation error in database), the presence of artifacts in the generated prediction model and a lack of biological knowledge for the genes correlated with methane emissions results into the difficulty to associate estimates obtain here with a mechanistic function. For example, it is known that different methanogen species found in rumen samples carry most of the genes identified in this study. However, some specific methanogenic species will lack a specific gene or a subunit within an operon as described in Kaster et al. (2011). Therefore, some species have a different impact on the relative abundance of a specific subunit gene compare to others within the same operon and, in consequence, on the coefficient value obtained by PLS analysis.

Although it would be of further interest to identify to which organisms these genes belong to, this is beyond the scope of this paper and has to be addressed in substantial more detail using different methodologies to provide accurate results. In addition, phylogenetic association with the functional genes studied here is still challenging and was not carried out to avoid wrong conclusions. This decision was made based on the fact that new methanogens are still discovered (see Vanwonterghem et al., 2017) and not necessarily carrying all the genes involved in the methane synthesis pathway. Furthermore, different clades have been identified and were even within the same genus (e.g., *Methanobrevibacter* SGMT or RO clade) differently correlated with methane emissions in the same samples (Tapio et al., 2017). Specifically, *Methanobrevibacter* clade SGMT but not RO, was found more abundant in low emitters while genera within methylotrophic methanogens were enriched in high emitting cattle.

### Most important phylogenetic parameters impacting on methane emissions

Within the taxonomic parameters tested, factors directly associated with methanogens were confirmed to be robust biomarkers, especially the relative abundance of *Methanobrevibacter* genus. This genus is known to be the most dominant and active in the rumen (Hook et al., 2010; Henderson et al., 2015; Tapio et al., 2017; Wang et al., 2017) and is also associated with higher CH_4_ emissions as confirmed here. Using the Kraken database, a wider diversity of methanogens in the rumen was found compared to the results obtained using the Greengenes database. This confirms preliminary observations by Poulsen et al. (2013) and Henderson et al. (2015), and also highlights the importance of the reference database used to characterize metagenomics data (Siegwald et al., 2017).

Sun et al. (2012) confirmed that not all methanogens are active continuously in a methanogenic environment and suggested that the availability of substrates was an important cue for population growth. For example, *Methanocaldococcus* spp.*, Methanotorris* spp. and the methylotrophic methanogen *Methanohalophilus* spp. were three low abundance genera that were highly correlated with CH_4_ emissions and identified as robust biomarkers across different diets and breeds which contrasted with the result for the main methylotrophic methanogen Candidatus *Methanomethylophilus*. Moreover, the possibility to use these biomarkers offers an efficient and cheaper alternative to metatranscriptomics considered as more accurate tool to predict methane emissions compared to metagenomics (Shi et al., 2014; Wallace et al., 2017). Finally, the identification of low abundance methanogen populations but not all the most abundant as robust biomarkers may also explain weaker correlations found between total methanogens and CH_4_ emissions when 16S rRNA or *mcrA* genes were targeted by qPCR (Mosoni et al., 2011; Morgavi et al., 2012). On the other hand, this weak correlation can also be the result of methane oxidation by methanotrophs. This study is one of the first confirming a greater abundance of methanotrophic populations, especially *Methylomonas* genus in rumen and being significantly negatively correlated with CH_4_ emissions. Genes associated with methanotrophy were not identified in this study and previously in the set of eight animals (2011 experiment) as highlighted by Wallace et al. (2015) and could be explained by not enough depth of sequencing for genes carried by very low abundant populations (0.1%). The genus *Methylomonas* is identified in Greengenes and Kraken databases but the last one contains a broader diversity of recently discovered microbial populations that could improve the detection of low abundance genus in rumen sample.

In terms of data directly associated with the microbial community composition, the Archaea:Bacteria ratio was confirmed as a strong biomarker of CH_4_ emissions while a lower R-value (*R* = 0.272) was found in this study compared to Wallace et al. paper (2014) which calculated this ratio on a reduced number of cattle (*R* = 0.49). This difference can be explained by the initial set of eight samples representing extreme methane emitters while the other 42 samples were not specifically selected for this trait. However, as also reported by the same authors, this significant correlation was diet dependent, and was significant for concentrate fed rumen samples but not forage samples. As previously shown in sheep by Kittelmann et al. (2014), the microbial community composition (PCoA-2 in this study) even at the phylum level was confirmed as robust biomarkers of CH_4_ emissions. This could be explained by an increase in the relative abundance of several bacterial populations within Firmicutes, Bacteroidetes, and Proteobacteria, mostly in low emitters as shown by the L/H ratio in Table 1. However, our study confirmed the necessity to calculate the methanogen diversity as robust biomarker instead of total microbial diversity, not significantly different between methane emitter groups in this study. These results differed from the idea developed by Shabat et al. (2016) that cattle with higher CH_4_ emissions will have higher total microbiome diversity.

### Link between microbial communities and metabolites released

In term of identifying links between the bacterial community containing most of the organic matter degraders and the metabolites released in rumen, it seems that the degradation activities carried out by the two most abundant bacterial phyla, Firmicutes and Bacteroidetes as evaluated using the F:B ratio (Chen et al., 2016) were not important to explain CH_4_ emissions. More interestingly, this study confirmed the importance of other bacterial populations associated with production of different metabolites which directly impacted on CH_4_ emissions and also showing a higher VIP value compared to methanogens (Table 2). For example, *Butyrivibrio* spp. and *Pseudobutyrivibrio* spp. both butyrate-producing bacteria were highly correlated with high CH_4_ emissions while the presence of bacteria metabolizing lactate (e.g., *Megasphaera*), degrading amino acids (e.g., *Acidaminococcus*) or competing for H_2_ were negatively correlated with CH_4_ emissions (Park et al., 2014; Kamke et al., 2016; Sa et al., 2016). This result is explained by the different catabolic pathways carried by these populations and directly impacting on H_2_ partial pressure and subsequently on CH_4_ emissions (Janssen, 2010; Kelly et al., 2010; Kamke et al., 2016; Sa et al., 2016; Tapio et al., 2017). The presence of lactate-utilizing *Megasphaera* genus within the robust phylogenetic biomarkers and negatively correlated with CH_4_ emissions, highlighted the importance of lactate metabolism controlling rumen fermentation (Counotte and Prins, 1981), production of H_2_ and specific VFAs and ultimately CH_4_ (Van Lingen et al., 2016). The impact that VFAs have on CH_4_ emissions is established (Janssen, 2010; Wanapat et al., 2015) and the positive correlation between different VFA or acetate-to-propionate ratio and CH_4_ emissions as previously stated by Shabat et al. (2016). However, none of the VFA factors were identified as strong biomarkers (Table 3) confirming some contrasting results found between VFA and CH_4_ emissions and reviewed in Negussie et al. (2017). It could be explained by necessity to study the relative inter-relationships among VFA measurements and also between VFA and CH_4_ yield as suggested by Palarea-Albaladejo et al. (2017). Therefore, the impact that VFA have on CH_4_ emissions may be less important compared to lactate metabolism and new strategies for methane mitigation could be developed based on this finding (Jeyanathan et al., 2014).

Genera within *Succinovibrionaceae* known to be dominant in the digestive tract of the Tammar wallaby, which emit one quarter of the methane emissions of the cattle (Pope et al., 2011) were not identified within low emitters as previously shown by Wallace et al. (2015). At the family level, the relative abundance of *Succinovibrionaceae* was 1.6-fold higher in low CH_4_ emitters (on average 1.3 ± 0.2) compared to high emitters (on average 0.8 ± 0.1) but associated with a weak significance level (*P* = 0.049). Surprisingly, these bacterial populations were not identified as robust biomarkers, probably because of the functional redundancy associated with the production or degradation of each metabolite. On the other hand, the *Opitutus* genus was characterized as a robust biomarker and is known to be involved in H_2_ production during the fermentation of organic matter (Chin et al., 2001). Very little information exists that explains the role of the *Dorea, Isosphaera, Faecalitalea, Colwellia*, and *Singulisphaera* on CH_4_ emissions but some were associated with degradation capacities in methane emitting environment (Kleindienst et al., 2016).

Finally, we agree that other potential biomarkers of CH_4_ emissions like archaeol could be tested (McCartney et al., 2013) and compared with the robust biomarkers identified in this study. The same authors showed the benefit of using archaeol over qPCR method as a proxy for CH_4_ emissions.

To the best of our knowledge, this is the first report identifying and comparing potential CH_4_ biomarkers across a range of dietary conditions and several experiments. This study confirms the possible value of targeting functional genes using metagenomics as most of the robust biomarkers identified were genes directly involved in the hydrogenotrophic methane synthesis pathway while methylotrophic methanogens were also important in explaining CH_4_ emissions. In addition, most of the genes directly involved in the methane synthesis pathway grouped in the same cluster within a functional genes network and this result was reproduced over three independent trials. Finally, this study confirm the significance of using robust and applicable biomarkers from the microbiome as a proxy of CH_4_ emissions across diverse beef cattle breeds fed with different diets as an alternative for a trait that is difficult-to-measure on a large number of animals. Moreover, the use of these biomarkers for the development of molecular tools will help for the implementation of breeding strategies targeting low-methane emitter animals.

## Author contributions

MA and RR: Conceptualization; MA and MW: Formal analysis; MA and RR: Original writing; MA, RD, C-AD, JR, RW, TF, RS, MW and RR: Review and Editing; All authors read and approved the final manuscript.

### Conflict of interest statement

The authors declare that the research was conducted in the absence of any commercial or financial relationships that could be construed as a potential conflict of interest.

## References

[B1] Borrel G. O'Toole P. W. Harris H. M. B. Peyret P. Brugère J.-F. Gribaldo S. (2013). Phylogenomic data support a seventh order of methylotrophic methanogens and provide insights into the evolution of methanogenesis. Genome Biol. Evol. 5, 1769–1780. 10.1093/gbe/evt128 23985970 PMC3814188

[B2] Buan N. R. Metcalf W. W. (2010). Methanogenesis by *Methanosarcina acetivorans* involves two structurally and functionally distinct classes of heterodisulfide reductase. Mol Microbiol. 75, 843–853. 10.1111/j.1365-2958.2009.06990.x 19968794

[B3] Chen S. Cheng H. Wyckoff K. N. He Q. (2016). Linkages of Firmicutes and Bacteroidetes populations to methanogenic process performance. J Ind Microbiol Biotechnol. 43, 771–781. 10.1007/s10295-016-1760-8 27021844

[B4] Chin K.-J. Liesack W. Janssen P. H. (2001). *Opitutus terrae* gen. nov., sp. nov., to accommodate novel strains of the division ‘Verrucomicrobia’ isolated from rice paddy soil. Int. J. Syst. Evol. Microbiol. 51, 1965–1968. 10.1099/00207713-51-6-1965 11760935

[B5] Cottle D. J. Nolan J. V. Wiedemann S. G. (2011). Ruminant enteric methane mitigation: a review. Anim. Prod. Sci. 51, 491–514. 10.1071/AN10163

[B6] Counotte G. H. Prins R. A. (1981). Regulation of lactate metabolism in the rumen. Vet. Res. Commun. 5, 101–115. 10.1007/BF02214975 7048723

[B7] Danielsson R. Dicksved J. Sun L. Gonda H. Müller B. Schnürer A.. (2017). Methane production in dairy cows correlates with rumen methanogenic and bacterial community structure. Front. Microbiol. 8:226. 10.3389/fmicb.2017.00226 28261182 PMC5313486

[B8] Desantis T. Z. Hugenholtz P. Larsen N. Rojas M. Brodie E. L. Keller K. (2006). Greengenes, a chimera-checked 16S rRNA gene database and workbench compatible with ARB. Appl. Environ. Microbiol. 72, 5069–5072. 10.1128/AEM.03006-05 16820507 PMC1489311

[B9] Duthie C. A. Haskell M. Hyslop J. J. Waterhouse A. Wallace R. J. Roehe R.. (2017). The impact of divergent breed types and diets on methane emissions, rumen characteristics and performance of finishing beef cattle. Animal 11, 1–10. 10.1017/S175173111700146X 28222832

[B10] Duthie C. A. Rooke J. A. Troy S. Hyslop J. J. Ross D. W. Waterhouse A.. (2016). Impact of adding nitrate or increasing the lipid content of two contrasting diets on blood methaemoglobin and performance of two breeds of finishing beef steers. Animal 10, 786–795. 10.1017/S1751731115002657 26627142

[B11] Freeman T. C. Goldovsky L. Brosch M. Van Dongen S. Mazière P. Grocock R. J. (2007). Construction, visualisation, and clustering of transcription networks from microarray expression data. PLoS Comput. Biol. 3:e206. 10.1371/journal.pcbi.0030206 17967053 PMC2041979

[B12] Giger-Reverdin S. Sauvant D. (2000). Methane production in sheep in relation to concentrate feed composition from bibliographic data, in Sheep and Goat Nutrition: Intake, Digestion, Quality of Products and Rangelands Zaragoza, eds Ledin I. Morand-Fehr P. (Zaragoza: Ciheam-Iamz), 43–46.

[B13] Guyader J. Doreau M. Morgavi D. P. Gérard C. Loncke C. Martin C. (2016). Long-term effect of linseed plus nitrate fed to dairy cows on enteric methane emission and nitrate and nitrite residuals in milk. Animal 10, 1173–1181. 10.1017/S1751731115002852 27075614

[B14] Guyader J. Eugène M. Meunier B. Doreau M. Morgavi D. P. Silberberg M.. (2015). Additive methane-mitigating effect between linseed oil and nitrate fed to cattle. J. Anim. Sci. 93, 3564–3577. 10.2527/jas.2014-8196 26440025

[B15] Henderson G. Cox F. Ganesh S. Jonker A. Young W. Collaborators G.. (2015). Rumen microbial community composition varies with diet and host, but a core microbiome is found across a wide geographical range. Sci Rep. 5:14567. 10.1038/srep14567 26449758 PMC4598811

[B16] Herd R. M. Arthur P. F. Bird S. H. Donoghue K. A. Hegarty R. S. (2014). Genetic Variation for Methane Traits in Beef Cattle. Congress of Genetics Applied to Livestock Production Vancouver, BC.

[B17] Hino T. Shimada K. Maruyama T. (1994). Substrate preference in a strain of *Megasphaera elsdenii*, a ruminal bacterium, and its implications in propionate production and growth competition. Appl. Environ. Microbiol. 60, 1827–1831. 16349276 10.1128/aem.60.6.1827-1831.1994 PMC201569

[B18] Hook S. E. Wright A. D. Mcbride B. W. (2010). Methanogens:methane producers of the rumen and mitigation strategies. Archaea 2010:945785. 10.1155/2010/945785. 21253540 PMC3021854

[B19] Immig I. Demeyer D. Fiedler D. Van Nevel C. Mbanzamihigo L. (1996). Attempts to induce reductive acetogenesis into a sheep rumen. Arch. Tierernahr. 49, 363–370. 10.1080/17450399609381898 8988318

[B20] Intergovernmental Panel on Climate Change (IPCC) (2014). Climate Change 2014. Synthesis report 2014.

[B21] Janssen P. H. (2010). Influence of hydrogen on rumen methane formation and fermentation balances through microbial growth kinetics and fermentation thermodynamics. Anim. Feed Sci. Technol. 160, 1–22. 10.1016/j.anifeedsci.2010.07.002

[B22] Jeyanathan J. Martin C. Morgavi D. (2014). The use of direct-fed microbials for mitigation of ruminant methane emissions: a review. Animal 8, 250–261. 10.1017/S1751731113002085 24274095

[B23] Johnson K. A. Johnson D. E. (1995). Methane emissions from cattle. J Anim Sci. 73, 2483–2492. 10.2527/1995.7382483x 8567486

[B24] Kamke J. Kittelmann S. Soni P. Li Y. Tavendale M. Ganesh S.. (2016). Rumen metagenome and metatranscriptome analyses of low methane yield sheep reveals a Sharpea enriched microbiome characterised by lactic acid formation and utilisation. Microbiome 4:56. 10.1186/s40168-016-0201-2 27760570 PMC5069950

[B25] Kaster A. K. Goenrich M. Seedorf H. Liesegang H. Wollherr A. Gottschalk G. (2011). More than 200 genes required for methane formation from H2 and CO2 and energy conservation are present in *Methanothermobacter marburgensis* and *Methanothermobacter thermautotrophicus*. Archaea 2011:973848 10.1155/2011/973848 21559116 PMC3087415

[B26] Kelly W. J. Leahy S. C. Altermann E. Yeoman C. J. Dunne J. C. Kong Z. (2010). The Glycobiome of the rumen bacterium *Butyrivibrio proteoclasticus* B316T highlights adaptation to a polysaccharide-rich environment. PLoS ONE 5:e11942 10.1371/journal.pone.0011942 20689770 PMC2914790

[B27] Kittelmann S. Pinares-Patiño C. S. Seedorf H. Kirk M. R. Ganesh S. Mcewan J. C.. (2014). Two different bacterial community types are linked with the low-methane emission trait in sheep. PLoS ONE 9:e103171. 10.1371/journal.pone.0103171 25078564 PMC4117531

[B28] Kleindienst S. Grim S. Sogin M. Bracco A. Crespo-Medina M. Joye S. B. (2016). Diverse, rare microbial taxa responded to the Deepwater Horizon deep-sea hydrocarbon plume. ISME J. 10, 400–415. 10.1038/ismej.2015.121 26230048 PMC4737931

[B29] Knapp J. R. Laur G. L. Vadas P. A. Weiss W. P. Tricarico J. M. (2014). Invited review: enteric methane in dairy cattle production: quantifying the opportunities and impact of reducing emissions. J. Dairy Sci. 97, 3231–3261. 10.3168/jds.2013-7234 24746124

[B30] Leahy S. C. Kelly W. J. Altermann E. Ronimus R. S. Yeoman C. J. Pacheco D. M.. (2010). The genome sequence of the rumen methanogen *Methanobrevibacter ruminantium* reveals new possibilities for controlling ruminant methane emissions. PLoS ONE 5:e8926. 10.1371/journal.pone.0008926 20126622 PMC2812497

[B31] Li Y. Leahy S. C. Jeyanathan J. Henderson G. Cox F. Altermann E.. (2016). The complete genome sequence of the methanogenic archaeon ISO4-H5 provides insights into the methylotrophic lifestyle of a ruminal representative of the *Methanomassiliicoccales*. Stand. Genomic Sci. 11:59. 10.1186/s40793-016-0183-5 27602181 PMC5011839

[B32] Malmuthuge N. Guan L. L. (2017). Understanding host-microbial interactions in rumen: searching the best opportunity for microbiota manipulation. Microbiome 8:8. 10.1186/s40104-016-0135-3 28116074 PMC5244612

[B33] McCartney C. A. Bull I. D. Waters S. M. Dewhurst R. J. (2013). Technical note: comparison of biomarker and molecular biological methods for estimating methanogen abundance. J. Anim. Sci. 91, 5724–5728. 10.2527/jas.2013-6513 24146154

[B34] Morgavi D. P. Martin C. Jouany J. P. Ranilla M. J. (2012). Rumen protozoa and methanogenesis: not a simple cause-effect relationship. Br. J. Nutr. 107, 388–397. 10.1017/S0007114511002935 21762544

[B35] Mosoni P. Martin C. Forano E. Morgavi D. P. (2011). Long-term defaunation increases the abundance of cellulolytic ruminococci and methanogens but does not affect the bacterial and methanogen diversity in the rumen of sheep. J. Anim. Sci. 89, 783–791. 10.2527/jas.2010-2947 21346137

[B36] Moss A. Jouany J.-P. Newbold J. (2000). Methane production by ruminants: its contribution to global warming. Ann. Zootech. 49, 231–253. 10.1051/animres:2000119

[B37] Negussie E. de Haas Y. Dehareng F. Dewhurst R. J. Dijkstra J. Gengler N.. (2017). Invited review: large-scale indirect measurements for enteric methane emissions in dairy cattle: a review of proxies and their potential for use in management and breeding decisions. J. Dairy Sci. 100, 2433–2453. 10.3168/jds.2016-12030 28161178

[B38] Olijhoek D. W. Hellwing A. L. F. Brask M. Weisbjerg M. R. Højberg O. Larsen M. K.. (2015). Effect of dietary nitrate level on enteric methane production, hydrogen emission, rumen fermentation, and nutrient digestibility in dairy cows. J. Dairy Sci. 99, 6191–6205. 10.3168/jds.2015-10691 27236758

[B39] Palarea-Albaladejo J. Rooke J. A. Nevison I. M. Dewhurst R. J. (2017). Compositional mixed modeling of methane emissions and ruminal volatile fatty acids from individual cattle and multiple experiments. J. Anim. Sci. 95, 2467–2480. 10.2527/jas.2016.1339 28727067

[B40] Park J. Park S. Kim M. (2014). Anaerobic degradation of amino acids generated from the hydrolysis of sewage sludge. Environ. Technol. 35, 1133–1139. 10.1080/09593330.2013.863951 24701908

[B41] Parmar N. R. Nirmal Kumar J. I. Joshi C. G. (2015). Exploring dietdependent shifts in methanogen and methanotroph diversity in the rumen of Mehsani buffalo by a metagenomics approach. Front. Life Sci. 8, 371–378. 10.1080/21553769.2015.1063550

[B42] Pinares-Patiño C. S. Hickey S. M. Young E. A. Dodds K. G. Maclean S. Molano G.. (2013). Heritability estimates of methane emissions from sheep. Animal 7, 316–321. 10.1017/S1751731113000864 23739473 PMC3691003

[B43] Pope P. B. Smith W. Denman S. E. Tringe S. G. Barry K. Hugenholtz P.. (2011). Isolation of Succinivibrionaceae implicated in low methane emissions from Tammar wallabies. Science 333, 646–648. 10.1126/science.1205760 21719642

[B44] Poulsen M. Schwab C. Jensen B. B. Engberg R. M. Spang A. Canibe N.. (2013). Methylotrophic methanogenic Thermoplasmata implicated in reduced methane emissions from bovine rumen. Nat. Commun. 4:1428. 10.1038/ncomms2432 23385573

[B45] Roehe R. Dewhurst R. J. Duthie C.-A. Rooke J. A. Mckain N. Ross D. W.. (2016). Bovine host genetic variation influences rumen microbial methane production with best selection criterion for low methane emitting and efficiently feed converting hosts based on metagenomic gene abundance. PLoS Genet. 12:e1005846. 10.1371/journal.pgen.1005846 26891056 PMC4758630

[B46] Rooke J. A. Wallace R. J. Duthie C.-A. Mckain N. De Souza S. M. Hyslop J. J.. (2014). Hydrogen and methane emissions from beef cattle and their rumen microbial community vary with diet, time after feeding and genotype. Br. J. Nutr. 112, 398–407. 10.1017/S0007114514000932 24780126

[B47] Ross E. M. Moate P. J. Marett L. Cocks B. G. Hayes B. J. (2013). Metagenomic predictions: from microbiome to complex health and environmental phenotypes in humans and cattle. PLoS ONE 8:e73056. 10.1371/journal.pone.0073056 24023808 PMC3762846

[B48] Russell J. B. Wallace R. J. (1997). Energy-yielding and energy-consuming reactions, in The Rumen Microbial Ecosystem, 2nd Edn, eds Hobson P. J. Stewart C. S. (London: Blackie Acad. Profess), 246–282.

[B49] Sa J. H. Kwak G. H. Han K. Ahn D. Cho S. J. Lee J. D.. (2016). Inhibition of methane and natural gas hydrate formation by altering the structure of water with amino acids. Sci Rep. 6:31582. 10.1038/srep31582 27526869 PMC4985706

[B50] Shabat S. K. Sasson G. Doron-Faigenboim A. Durman T. Yaacoby S. Berg Miller M. E.. (2016). Specific microbiome-dependent mechanisms underlie the energy harvest efficiency of ruminants. ISME J. 10, 2958–2972. 10.1038/ismej.2016.62 27152936 PMC5148187

[B51] Shi W. Moon C. D. Leahy S. C. Kang D. Froula J. Kittelmann S.. (2014). Methane yield phenotypes linked to differential gene expression in the sheep rumen microbiome. Genome Res. 24, 1517–1525. 10.1101/gr.168245.113 24907284 PMC4158751

[B52] Siegwald L. Touzet H. Lemoine Y. Hot D. Audebert C. Caboche S. (2017). Assessment of common and emerging bioinformatics pipelines for targeted metagenomics. PLoS ONE 12:e0169563. 10.1371/journal.pone.0169563 28052134 PMC5215245

[B53] Sun C. L. Brauer S. L. Cadillo-Quiroz H. Zinder S. H. Yavitt J. B. (2012). Seasonal changes in methanogenesis and methanogenic community in three peatlands, New York State. Front. Microbiol. 3:81. 10.3389/fmicb.2012.00081 22408638 PMC3294236

[B54] Tapio I. Snelling T. J. Strozzi F. Wallace R. J. (2017). The ruminal microbiome associated with methane emissions from ruminant livestock. J. Anim. Sci. Biotechnol. 8:7. 10.1186/s40104-017-0141-0 28123698 PMC5244708

[B55] Terré M. Castells L. Fàbregas F. (2013). Short communication: comparison of pH, volatile fatty acids, and microbiome of rumen samples from preweaned calves obtained via cannula or stomach tube. J. Dairy Sci. 96, 5290–5294. 10.3168/jds.2012-5921 23706486

[B56] Thauer R. K. Kaster A.-K. Seedorf H. Buckel W. Hedderich R. (2008). Methanogenic archaea: ecologically relevant differences in energy conservation. Nat. Rev. Microbiol. 6, 579–591. 10.1038/nrmicro1931 18587410

[B57] Van Lingen H. J. Plugge C. M. Fadel J. G. Kebreab E. Bannink A. Dijkstra J. (2016). Thermodynamic driving force of hydrogen on rumen microbial metabolism: a theoretical investigation. PLoS ONE 11:e0161362 10.1371/journal.pone.0161362 27783615 PMC5081179

[B58] Vanwonterghem I. Evans P. N. Parks D. H. Jensen P. D. Woodcroft B. J. Hugenholtz P.. (2017). Methylotrophic methanogenesis discovered in the archaeal phylum Verstraetearchaeota. Nat. Microbiol. 1:16170. 10.1038/nmicrobiol.2016.170 27694807

[B59] Veneman J. B. Muetzel S. Hart K. J. Faulkner C. L. Moorby J. M. Perdok H. B. (2015). Does dietary mitigation of enteric methane production affect rumen function and animal productivity in dairy cows? PLoS ONE 10:e0140282. 10.1371/journal.pone.0140282 26509835 PMC4624802

[B60] Wallace R. J. Rooke J. A. Duthie C.-A. Hyslop J. J. Ross D. W. Mckain N.. (2014). Archaeal abundance in post-mortem ruminal digesta may help predict methane emissions from beef cattle. Sci Rep. 4:5892. 10.1038/srep05892 25081098 PMC5376199

[B61] Wallace R. J. Rooke J. A. Mckain N. Duthie C.-A. Hyslop J. J. Ross D. W.. (2015). The rumen microbial metagenome associated with high methane production in cattle. BMC Genomics 16:839. 10.1186/s12864-015-2032-0 26494241 PMC4619255

[B62] Wallace R. J. Snelling T. J. Mccartney C. A. Tapio I. Strozzi F. (2017). Application of meta-omics techniques to understand greenhouse gas emissions originating from ruminal metabolism. Genet. Sel. Evol. 16:9 10.1186/s12711-017-0285-6 PMC5240273 28093073

[B63] Wanapat M. Cherdthong A. Phesatcha K. Kang S. (2015). Dietary sources and their effects on animal production and environmental sustainability. Anim. Nutr. 1, 96–103. 10.1016/j.aninu.2015.07.004 PMC5945976 29767156

[B64] Wang Z. Elekwachi C. O. Jiao J. Wang M. Tang S. Zhou C.. (2017). Investigation and manipulation of metabolically active methanogen community composition during rumen development in black goats. Sci Rep. 7:442. 10.1038/s41598-017-00500-5 28341835 PMC5428682

[B65] Wold S. (1995). PLS for multivariate linear modelling, in Chemometric Methods in Molecular Design, ed Van de Waterbeemd H. (Weinheim: VCH Publishers), 195–218.

[B66] Wolin M. J. Miller T. L. Stewart C. S. (1997). Microbe–microbe interactions, in The Rumen Microbial Ecosystem, eds Hobson P. N. Stewart C. S. (London: Chapman and Hall), 467–491.

[B67] Wood D. E. Salzberg S. L. (2014). Kraken: ultrafast metagenomic sequence classification using exact alignments. Genome Biol. Evol. 15:R46. 10.1186/gb-2014-15-3-r46 24580807 PMC4053813

[B68] Yang C. Rooke J. A. Cabeza I. Wallace R. J. (2016). Nitrate and inhibition of ruminal methanogenesis: microbial ecology, obstacles, and opportunities for lowering methane emissions from ruminant livestock. Front. Microbiol. 7:132. 10.3389/fmicb.2016.00132 26904008 PMC4751266

