# Supplementary figures and images for: Identification, Comparison, and Validation of Robust Rumen Microbial Biomarkers for Methane Emissions Using Diverse Bos Taurus Breeds and Basal Diets
Source: Front Microbiol. 2018 Jan 9;8:2642. doi: 10.3389/fmicb.2017.02642 (PMC5767246; doi:10.3389/fmicb.2017.02642)

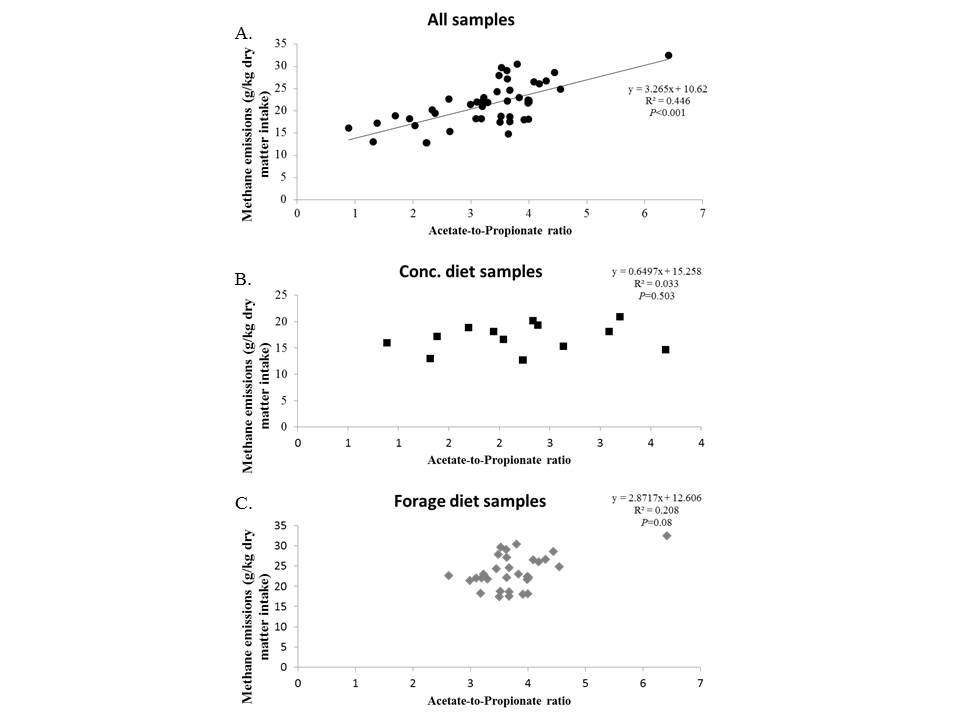

Supplement: Supplementary file 1 [file Image1.jpg]

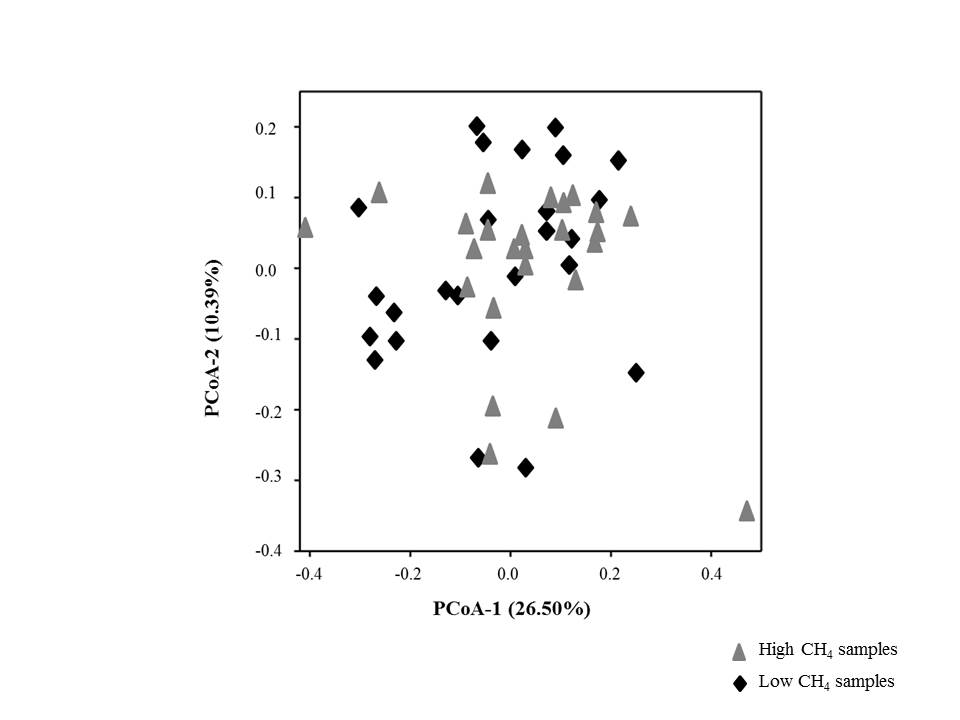

Supplement: Supplementary file 2 [file Image2.JPEG]

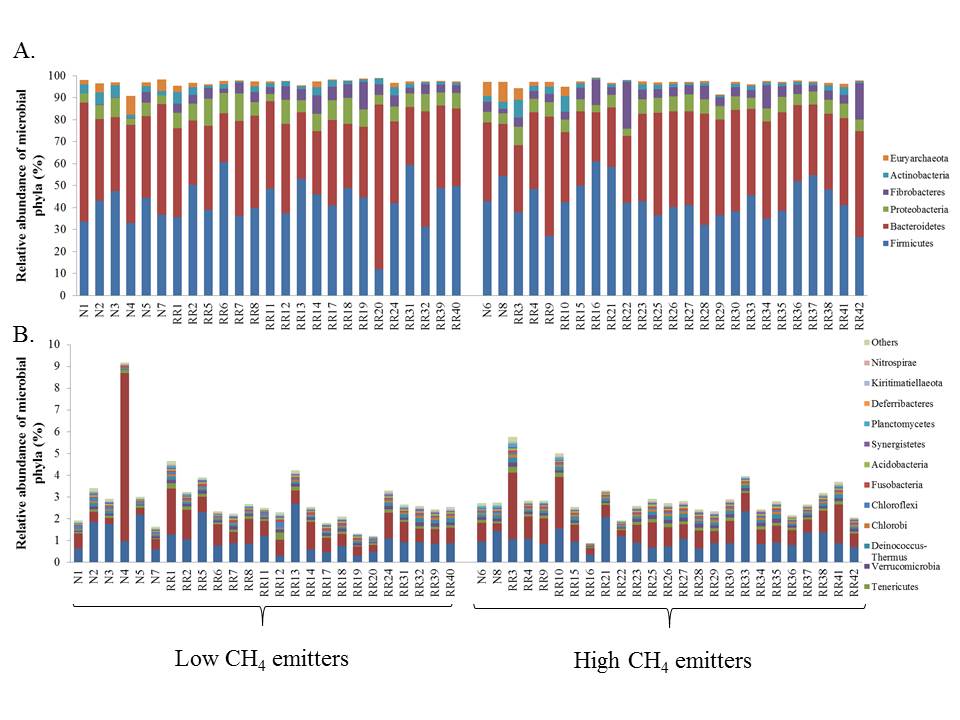

Supplement: Supplementary file 3 [file Image3.JPEG]

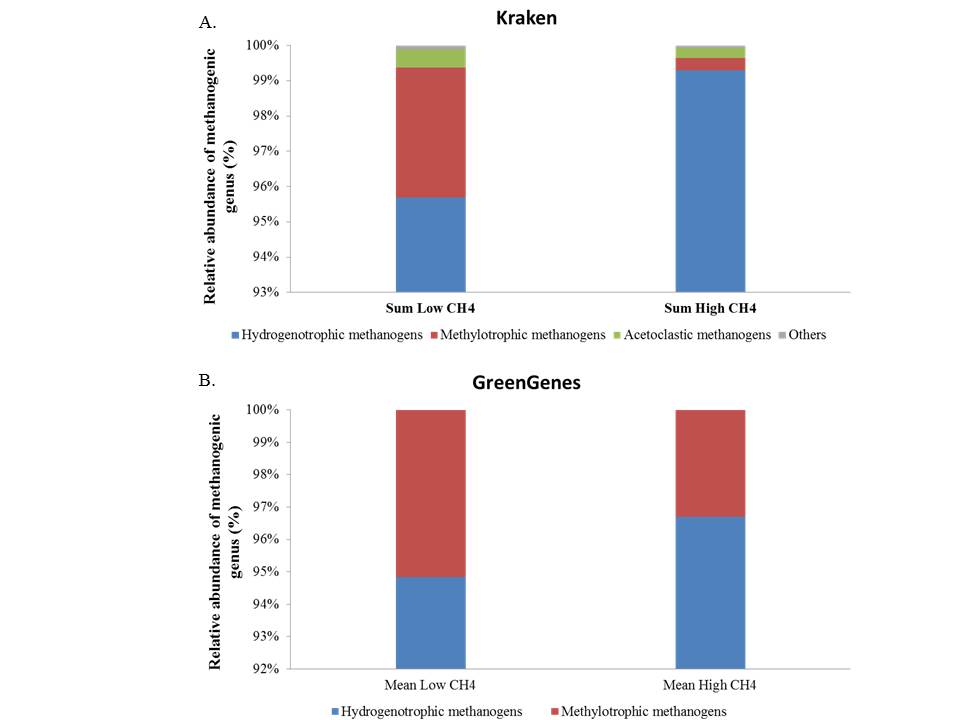

Supplement: Supplementary file 4 [file Image4.jpg]

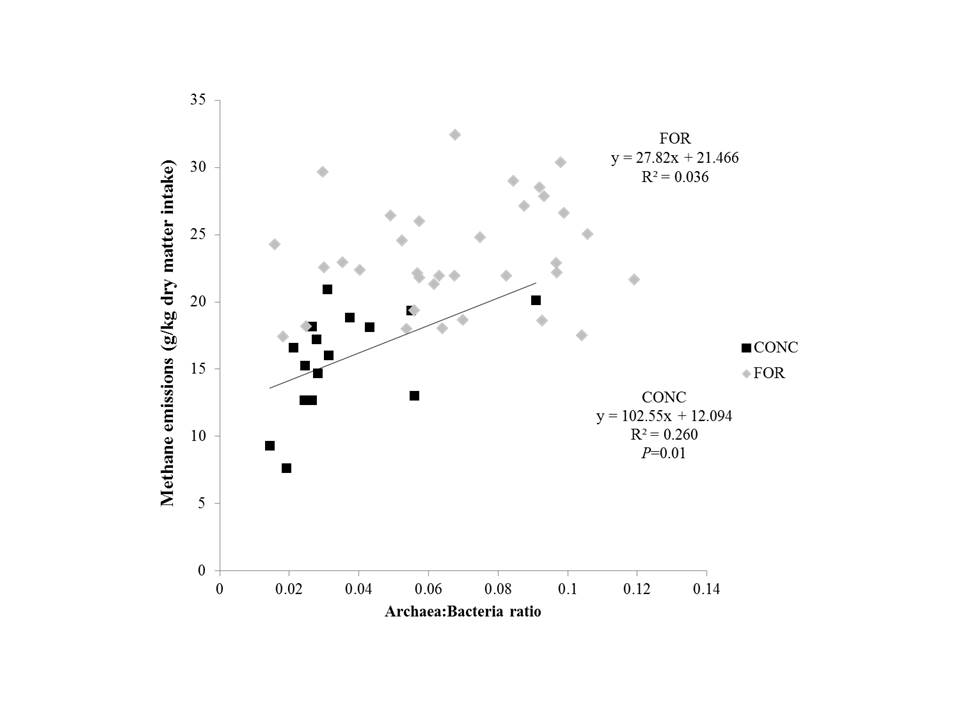

Supplement: Supplementary file 5 [file Image5.JPEG]
