# Supplementary material for: Identification, Comparison, and Validation of Robust Rumen Microbial Biomarkers for Methane Emissions Using Diverse Bos Taurus Breeds and Basal Diets
Source: Front Microbiol. 2018 Jan 9;8:2642. doi: 10.3389/fmicb.2017.02642 (PMC5767246; doi:10.3389/fmicb.2017.02642)
Supplement: Supplementary file 6 [file Presentation1.PDF]

Identification, comparison and validation of robust rumen microbial biomarkers of methane emissions from cattle across diverse breeds and diets

Marc D. Auffret*[1], Robert Stewart[2], Richard J. Dewhurst[1], Carol-Anne Duthie[1], John A. Rooke[1], R. John Wallace[3], Tom C. Freeman[4], Timothy Snelling[3], Mick Watson[2,4], Rainer Roehe[1].

* Corresponding author
[1] SRUC, Edinburgh, United Kingdom, [2] Edinburgh Genomics, The Roslin Institute and R(D)SVS, University of Edinburgh, Edinburgh, United Kingdom, [3] Rowett Institute, University of Aberdeen, Aberdeen, United Kingdom, [4] Division of Genetics and Genomics, The Roslin Institute and R(D)SVS, University of Edinburgh, Edinburgh, United Kingdom, [4] Edinburgh Genomics, The Roslin Institute and R(D)SVS, University of Edinburgh, Edinburgh, United Kingdom.

Correspondence:

Marc.Auffret@sruc.ac.uk

Supporting information

Figure S1: Linear regression between Acetate:Propionate ratio as a proxy of hydrogen availability in rumen and CH_4 emissions.
(A) All samples (n=50). (B) Samples from concentrate-fed animals (n=16). (C) Samples from forage-fed animals (n=34). Equation for the linear regression was included in figure when the difference was significant (*P*-value<0.05).

Figure S2: Principal Coordinates analysis (PCoA) of the microbial community structure identified at the phylum level (number of animals, n=50 samples). Black diamond: Low methane emitters, Grey triangle: High methane emitters.

Figure S3A: Microbial community composition of the most dominant populations at the phylum level (n=50).

Figure S3B: Microbial community composition of minor populations at the phylum level (n=50).
Others: *Aquificae*, *Armatimonadetes*, *Calditrichaeota*, *Chlamydiae*, *Chrysiogenetes*, *Crenarchaeota*, *Elusimicrobia*, Gemmatimonadetes, *Ignavibacteriae*, *Thaumarchaeota*, *Thermodesulfobacteria* and *Thermotogae*.

Figure S4: Diversity of methanogen synthesis pathway using (A) Kraken database or (B) Greengenes database.

Figure S5: Linear regression between Acetate:Propionate ratio as a proxy of hydrogen availability in rumen and CH_4 emissions.
All samples (n=50) including the distinction between forage-fed animals (n=34, grey diamonds) and concentrate-fed animals (n=16, black squares). Equation for the linear regression was included in figure when the difference was significant (*P*-value<0.05).

Table S1: Sample characteristics and volatile fatty acid molar proportions (mmol/mol) measured on slaughter rumen samples.

**Year-Sample**	**Breed type**	**Diet**	**Methane (g/kg DMI)**	**Acetate**	**Propionate**	**Isobutyrate**	**Butyrate**	**Isovalerate**	**Valerate**	**A:P ratio[1]**
2011-N1*	AA sired	CONC-CONT	7.63	N.D.	N.D.	N.D.	N.D.	N.D.	N.D.	N.D.
2011-N2*	AA sired	CONC-CONT	18.14	599	194	17	138	32	19	3.09
2011-N3*	Lim. sired	CONC-CONT	9.29	N.D.	N.D.	N.D.	N.D.	N.D.	N.D.	N.D.
2011-N4*	Lim. sired	CONC-CONT	20.13	567	243	15	125	33	17	2.33
2011-N5*	AA sired	FOR-CONT	17.41	665	190	11	107	14	13	3.50
2011-N6*	AA sired	FOR-CONT	32.42	725	113	10	123	15	13	6.42
2011-N7*	Lim. sired	FOR-CONT	19.37	N.D.	N.D.	N.D.	N.D.	N.D.	N.D.	N.D.
2011-N8*	Lim. sired	FOR-CONT	30.37	646	170	11	145	16	13	3.80
2013-RR1	LU sired	FOR-NIT	17.96	660.1	168.6	12.0	131.2	14.3	13.8	3.91
2013-RR2	LU sired	FOR-NIT	18.16	658.6	207.1	10.9	94.4	13.0	15.9	3.18
2013-RR3	CH sired	FOR-NIT	25.97	664.0	158.8	19.1	120.5	22.1	15.5	4.18
2013-RR4	CH sired	FOR-NIT	22.93	645.1	200.0	11.1	115.9	13.2	14.6	3.23
2013-RR5	LU sired	CONC-NIT	18.85	530.9	313.3	12.6	102.4	19.4	21.4	1.69
2013-	CH sired	CONC-	20.93	605.7	189.5	8.8	164.3	15.1	16.4	3.20

RR6		NIT								
2013-RR7	CH sired	CONC-NIT	12.70	590.4	264.2	19.0	76.8	27.3	22.3	2.23
2013-RR8	LU sired	CONC-NIT	16.61	554.4	272.0	9.5	116.7	16.7	30.6	2.04
2013-RR9	LU sired	FOR-CONT	29.64	684.2	193.8	9.7	86.2	11.2	14.9	3.53
2013-RR10	CH sired	FOR-CONT	27.12	668.0	184.0	12.0	105.8	16.8	13.4	3.63
2013-RR11	CH sired	CONC-CONT	15.28	561.5	213.1	19.3	162.5	25.3	18.4	2.64
2013-RR12	CH sired	CONC-CONT	16.01	401.2	452.5	3.0	110.1	6.1	27.1	0.89
2013-RR13	LU sired	CONC-CONT	18.16	567.4	292.1	20.4	74.8	25.5	19.8	1.94
2013-RR14	LU sired	CONC-CONT	19.36	602.0	252.9	18.8	83.3	23.3	19.7	2.38
2013-RR15	LU sired	FOR-CONT	28.99	666.5	184.2	17.1	101.6	18.6	11.9	3.62
2013-RR16	CH sired	FOR-CONT	24.25	661.3	191.5	5.0	115.6	7.7	18.9	3.45
2013-RR17	LU sired	CONC-RSC	12.71	604.0	270.7	19.3	66.4	15.8	23.9	2.23
2013-RR18	CH sired	CONC-RSC	13.00	508.2	386.1	11.8	56.1	16.2	21.6	1.32
20123-RR19	CH sired	CONC-RSC	14.69	654.0	179.4	9.9	130.4	15.8	10.6	3.65
2013-RR20	LU sired	CONC-RSC	17.20	502.3	363.3	12.3	79.1	16.7	26.2	1.38
2013-RR21	CH sired	FOR-RSC	21.92	620.9	200.1	11.1	136.0	14.6	17.3	3.10
2013-	CH sired	FOR-	22.54	636.4	242.8	9.8	79.0	15.5	16.5	2.62

RR22		RSC								
2013-RR23	LU sired	FOR-RSC	21.79	677.6	205.9	11.2	76.1	15.2	14.0	3.29
2013-RR24	LU sired	FOR-RSC	18.60	660.9	179.9	10.8	114.6	18.1	15.8	3.67
2014-RR25	Lim. sired	FOR-CONT	28.53	656.8	147.9	15.9	155.9	14.3	9.2	4.44
2014-RR26	Lim. sired	FOR-RSC	26.59	667.6	155.3	10.3	146.3	11.2	9.3	4.30
2014-RR27	Lim. sired	FOR-Comb	21.94	675.2	168.6	10.2	127.2	10.3	8.4	4.00
2014-RR28	AA sired	FOR-RSC	22.17	686.0	171.4	9.1	108.7	11.8	13.1	4.00
2014-RR29	AA sired	FOR-NIT	24.81	655.0	144.0	5.0	176.2	9.2	10.6	4.55
2014-RR30	Lim. sired	FOR-NIT	22.91	652.1	170.2	11.5	139.3	12.5	14.4	3.83
2014-RR31	AA sired	FOR-Comb	17.51	647.9	176.6	16.1	129.2	11.9	18.3	3.67
2014-RR32	AA sired	FOR-CONT	21.33	646.2	216.1	9.1	100.2	13.9	14.5	2.99
2014-RR33	AA sired	FOR-Comb	21.94	655.9	205.5	7.6	111.5	9.2	10.2	3.19
2014-RR34	AA sired	FOR-CONT	22.36	680.6	170.8	14.3	116.0	9.3	9.0	3.98
2014-RR35	Lim. sired	FOR-CONT	22.10	684.9	188.5	9.0	93.4	12.8	11.3	3.63
2014-RR36	AA sired	FOR-RSC	26.42	673.8	164.7	10.3	129.8	10.6	10.8	4.09
2014-RR37	Lim. sired	FOR-Comb	21.64	671.4	168.4	8.7	126.5	15.0	10.0	3.99
2014-	AA sired	FOR-	27.83	651.0	187.0	11.1	124.2	13.4	13.2	3.48

RR38		NIT								
2014-RR39	Lim. sired	FOR-NIT	18.65	658.2	186.9	12.3	115.0	14.0	13.7	3.52
2014-RR40	AA sired	FOR-Comb	18.04	662.8	165.8	13.8	130.3	15.6	11.6	4.00
2014-RR41	Lim. Sired	FOR-Comb	25.03	N.D.	N.D.	N.D.	N.D.	N.D.	N.D.	N.D.
2014-RR42	Lim. sired	FOR-RSC	24.53	678.9	184.9	14.0	105.1	7.5	9.6	3.67

Samples studied in Roehe et al. (2016), N.D.: Not Determined. [1]Acetate-to-Propionate ratio, [2]Correlation with CH_4 emissions.$P<0.05$

Table S2: Microbial phyla significantly different between conditions

Factor	Comparison LOW/HIGH		Comparison CONC/FOR		Comparison between all additives treatments within basal diet						
	**Mean LOW**	**Mean HIGH**	**Mean CONC**	**Mean FOR**	**Mean CONC -Cont**	**Mean CONC-NIT**	**Mean CONC -RSC**	**Mean FOR-Cont**	**Mean FOR-NIT**	**Mean FOR-RSC**	**Mean FOR-Comb**
Acidobacteria	0.059	0.059	0.053	0.058	0.048	0.060	0.055	0.055	0.063	0.056	0.063
Actinobacteria	2.168	2.219	2.525	2.334	3.238	1.604	2.022	2.489	3.157	1.701	1.773
Aquificae	0.011	0.013	0.010	0.013	0.014	0.005	0.008	0.011	0.015	0.012	0.015
Armatimonadetes	0.002	0.001	0.002	0.002	0.004	0.003	0.000	0.003	0.004	0.000	0.000
Bacteroidetes	37.852	38.942	39.388	38.281	38.665	36.396	43.827	39.829	37.168	38.872	35.880
Calditrichaeota	0.005*	0.001	0.006**	0.002	0.006	0.003	0.008	0.001	0.003	0.001	0.004
Chlamydiae	0.011	0.01	0.010	0.010	0.010	0.010	0.010	0.009	0.011	0.010	0.009
Chlorobi	0.06	0.08*	0.046	0.079*	0.049	0.047	0.039	0.075	0.087	0.074	0.082
Chloroflexi	0.069	0.052	0.088*	0.055	0.125*	0.052	0.052	0.058	0.064	0.051	0.043
Chrysiogenetes	0.002	0.004	0.003	0.005	0.005	0.000	0.000	0.007	0.003	0.004	0.002
Crenarchaeota	0.006	0.007	0.004	0.007	0.006	0.005	0.000	0.006	0.010	0.006	0.006
Cyanobacteria	0.851	0.977	1.106	0.943	1.551	0.833	0.491	0.818	1.353	0.735	0.924
Deferribacteres	0.015	0.017	0.011	0.017	0.012	0.010	0.010	0.015	0.019	0.018	0.017
Deinococcus-Thermus	0.102	0.135**	0.078	0.135°	0.083	0.081	0.065	0.128	0.146	0.118	0.157
Elusimicrobia	0.011	0.013	0.011	0.012	0.012	0.016	0.005	0.013	0.011	0.012	0.013
Euryarchaeota	1.024	1.193	1.644	1.743	2.579	1.131	0.285	2.733	1.822	0.861	0.832
Fibrobacteres	5.052	5.964	4.306	5.175	2.826	4.098	7.474	4.888	3.955	8.113	3.456

Firmicutes	42.525	42.981	41.536	43.325	42.794	43.970	36.588	41.686	43.011	42.116	48.637
Fusobacteria	0.073	0.067	0.069	0.065	0.065	0.073	0.073	0.057	0.072	0.066	0.069
Gemmatimonadetes	0.011	0.011	0.010	0.011	0.010	0.010	0.010	0.009	0.012	0.011	0.011
Ignavibacteriae	0.005	0.006	0.003	0.006	0.005	0.000	0.003	0.006	0.008	0.003	0.009
Kiritimatiellaeota	0.013	0.016*	0.008	0.017*	0.007	0.010	0.008	0.015	0.020	0.012	0.020
Nitrospirae	0.013	0.012	0.013	0.012	0.016	0.010	0.010	0.011	0.014	0.014	0.011
Planctomycetes	0.033	0.037	0.028	0.041	0.034	0.031	0.013	0.045	0.059	0.025	0.034
Proteobacteria	7.833*	6.025	7.689	6.072	6.275	10.013*	8.192	5.699	6.705	5.774	6.371
Spirochaetes	0.977	1.046	1.050	1.065	1.253	1.198	0.497	1.014	1.054	1.015	1.246
Synergistetes	0.058	0.057	0.054	0.056	0.049	0.063	0.057	0.052	0.058	0.058	0.056
Tenericutes	0.13	0.127	0.123	0.126	0.148	0.115	0.081	0.119	0.161	0.115	0.110
Thaumarchaeota	0.001	0.000	0.002	0.001	0.003	0.000	0.003	0.001	0.001	0.000	0.000
Thermodesulfobacteria	0.012	0.01	0.011	0.010	0.007	0.016	0.016	0.008	0.010	0.011	0.011
Thermotogae	0.01	0.011	0.013	0.012	0.017	0.013	0.003	0.014	0.018	0.007	0.009
Verrucomicrobia	0.122	0.132*	0.097	0.130	0.084	0.123	0.096	0.124	0.140	0.130	0.130
Firmicutes: Bacteroidetes ratio	1.295	1.184	1.177	1.390	1.067	1.203	1.197	1.174	1.422	1.177	1.390

**$P<0.01$, *$P<0.05$.

Table S3: PLS results identifying the most important genes directly involved in the methane emissions pathway.

KEGG ID	Function	VIP	Coefficient
K00123	formate dehydrogenase, alpha subunit	0.89	0.02
K00125	formate dehydrogenase, beta subunit	1.00	-0.03
K00169	pyruvate ferredoxin oxidoreductase, alpha subunit	0.97	0.05
K00170	pyruvate ferredoxin oxidoreductase, beta subunit	0.94	0.04
K00171	pyruvate ferredoxin oxidoreductase, delta subunit	0.97	0.07
K00200	formylmethanofuran dehydrogenase subunit A	1.00	0.06
K00201	formylmethanofuran dehydrogenase subunit B	0.92	0.05
K00202	formylmethanofuran dehydrogenase subunit C	1.00	-0.03
K00203	formylmethanofuran dehydrogenase subunit D	1.02	0.07
K00204	formylmethanofuran dehydrogenase subunit H	0.82	0.02
K00205	formylmethanofuran dehydrogenase subunit F	0.86	0.04
K00399	methyl-coenzyme M reductase alpha subunit	0.85	0.02
K00400	methyl coenzyme M reductase system, component A2	0.94	0.04
K00401	methyl-coenzyme M reductase beta subunit	0.97	-0.03
K00402	methyl-coenzyme M reductase gamma subunit	0.82	0.01
K03422	methyl-coenzyme M reductase subunit D	0.88	-0.04
K00440	coenzyme F420 hydrogenase alpha subunit	1.18	-0.08
K00441	coenzyme F420 hydrogenase beta subunit	0.89	-0.01
K00442	coenzyme F420 hydrogenase delta subunit	0.70	-0.01
K00443	coenzyme F420 hydrogenase gamma subunit	0.95	-0.04
K00577	tetrahydromethanopterin S-methyltransferase subunit A	0.93	-0.01
K00579	tetrahydromethanopterin S-methyltransferase subunit C	0.80	0.02
K00580	tetrahydromethanopterin S-methyltransferase subunit D	0.95	0.05
K00581	tetrahydromethanopterin S-methyltransferase subunit E	0.86	0.01
K00584	tetrahydromethanopterin S-methyltransferase subunit H	1.06	0.08
K00672	formylmethanofuran--tetrahydromethanopterin N-formyltransferase	0.87	0.01
K01499	methenyltetrahydromethanopterin cyclohydrolase	0.98	0.06
K13812	bifunctional enzyme Fae/Hps	0.94	0.04
K13942	5,10-methenyltetrahydromethanopterin hydrogenase	0.82	0.03
K14115	energy-converting hydrogenase B subunit F	0.86	0.01
K14120	energy-converting hydrogenase B subunit K	0.94	0.08
K14121	energy-converting hydrogenase B subunit L	0.82	-0.04
K14122	energy-converting hydrogenase B subunit M	0.83	0.04
K14123	energy-converting hydrogenase B subunit N	1.06	-0.05
K14126	F420-non-reducing hydrogenase subunit A	1.02	-0.04
K14128	F420-non-reducing hydrogenase subunit G	0.94	0.05

Table S4: Summary of the potential biomarkers associated with CH_4 emissions.

**Sample**	**Corrected CH_4[1]**	**PCoA-1**	**PCoA-2**	**Total Diversity[2]**	**Total Evenness[2]**	**Methanogens Diversity[2]**	**Methanogens Evenness[2]**	**Proteobacteria**	**F:B**	**A:B**
N1	N.D.	-0.272	-0.132	1.201	0.319	0.776	0.222	4.176	0.621	0.019
N2	0.430	0.028	-0.283	1.445	0.384	0.380	0.108	6.396	1.163	0.043
N3	N.D.	-0.061	-0.265	1.351	0.359	1.104	0.313	8.874	1.411	0.014
N4	0.632	-0.283	-0.099	1.530	0.407	0.261	0.074	2.991	0.738	0.091
N5	-1.575	-0.269	-0.042	1.389	0.369	0.406	0.115	6.126	1.193	0.018
N6	2.755	-0.037	-0.203	1.511	0.399	0.242	0.069	4.787	1.197	0.068
N7	N.D.	-0.230	-0.103	1.231	0.325	0.213	0.061	3.878	0.728	0.056
N8	1.691	-0.041	-0.271	1.419	0.375	0.216	0.061	4.817	2.291	0.098
RR1	-1.432	0.241	-0.150	1.767	0.467	0.448	0.127	6.869	0.883	0.054
RR2	-1.375	-0.042	-0.103	1.581	0.418	0.517	0.147	7.699	1.716	0.025
RR3	0.826	0.465	-0.348	1.966	0.520	0.273	0.078	8.366	1.231	0.057
RR4	-0.016	-0.086	-0.033	1.513	0.400	0.388	0.110	6.149	1.381	0.035
RR5	0.615	0.103	0.159	1.574	0.416	0.460	0.130	12.269	1.022	0.037
RR6	1.222	-0.104	-0.038	1.383	0.365	0.750	0.213	9.258	2.755	0.031
RR7	-1.079	0.084	0.196	1.468	0.388	0.407	0.116	12.509	0.836	0.027
RR8	-0.005	-0.123	-0.033	1.527	0.403	0.856	0.243	6.015	0.948	0.021
RR9	1.893	-0.035	-0.062	1.527	0.404	0.329	0.093	6.460	0.501	0.030
RR10	1.155	0.090	-0.220	1.837	0.486	0.353	0.100	5.803	1.329	0.088
RR11	-0.355	-0.237	-0.060	1.334	0.353	0.608	0.172	3.240	1.221	0.024
RR12	-0.154	0.216	0.153	1.512	0.400	0.542	0.154	11.032	0.920	0.031
RR13	0.424	0.073	0.052	1.451	0.384	0.278	0.079	5.556	1.744	0.027
RR14	0.757	-0.045	0.067	1.648	0.435	0.510	0.145	7.935	1.600	0.055
RR15	1.692	0.027	0.020	1.552	0.410	0.271	0.077	5.398	1.467	0.084
RR16	0.349	-0.409	0.052	1.200	0.319	0.282	0.080	3.273	2.692	0.016
RR17	-1.093	-0.056	0.180	1.455	0.384	0.643	0.184	8.837	1.051	0.024
RR18	-0.992	0.026	0.167	1.506	0.398	0.953	0.270	11.743	1.671	0.056
RR19	-0.518	-0.063	0.199	1.459	0.386	0.452	0.128	7.806	1.388	0.028

RR20	0.158	-0.307	0.084	1.059	0.281	0.254	0.072	4.382	0.159	0.028
RR21	-0.295	-0.072	0.023	1.468	0.388	0.231	0.066	5.671	2.168	0.063
RR22	-0.124	-0.262	0.101	1.457	0.385	0.293	0.083	3.413	1.389	0.030
RR23	-0.346	0.023	0.039	1.572	0.415	0.232	0.066	6.738	1.083	0.057
RR24	-1.247	0.120	0.004	1.739	0.460	0.393	0.111	6.789	1.131	0.093
RR25	1.150	0.172	0.031	1.585	0.419	0.248	0.070	6.838	0.783	0.092
RR26	0.606	0.175	0.039	1.571	0.415	0.247	0.070	6.891	0.929	0.099
RR27	-0.669	0.171	0.072	1.547	0.409	0.204	0.058	7.751	0.970	0.067
RR28	-0.242	0.122	0.095	1.554	0.411	0.307	0.087	6.575	0.637	0.097
RR29	0.472	0.109	0.085	1.485	0.392	0.235	0.067	6.050	0.842	0.075
RR30	-0.403	0.103	0.048	1.586	0.419	0.381	0.108	6.170	0.828	0.097
RR31	-1.546	0.007	-0.012	1.516	0.400	0.898	0.255	6.021	2.261	0.104
RR32	-0.470	0.177	0.096	1.487	0.393	0.273	0.078	8.037	0.597	0.062
RR33	-0.304	0.011	0.022	1.591	0.421	0.360	0.102	5.184	1.161	0.082
RR34	-0.190	0.081	0.089	1.548	0.411	0.298	0.085	5.981	0.795	0.040
RR35	-0.625	0.238	0.066	1.540	0.407	0.170	0.048	6.988	0.862	0.057
RR36	0.916	-0.088	0.058	1.428	0.377	0.207	0.059	4.966	1.496	0.049
RR37	-0.754	-0.046	0.049	1.484	0.392	0.289	0.082	5.878	1.703	0.119
RR38	1.314	0.031	-0.001	1.610	0.426	0.279	0.079	6.366	1.389	0.093
RR39	-1.613	0.076	0.081	1.461	0.389	0.760	0.216	5.969	1.303	0.070
RR40	-1.391	0.119	0.042	1.497	0.396	0.540	0.153	6.870	1.402	0.064
RR41	N.D.	0.128	-0.025	1.718	0.454	0.233	0.066	6.519	1.038	0.106
RR42	0.041	-0.045	0.116	1.540	0.407	0.318	0.090	5.146	0.555	0.053

[1]Methane emissions were corrected to remove the effect of diets and breeds on the index. [2]Shannon diversity index. N.D.: Not determined because of missing data for VFA.

Table S4 (suite): Summary of the potential biomarkers associated with CH_4 emissions

Sample	Euryarchaeota	Total Methanogens	*Methanobrevibacter*	*Methanohalophilus*	Total Methanotrophs	*Methylomonas*
N1	1.880	2.002	1.668	0.003	0.051	0.012
N2	4.134	4.947	4.715	0.002	0.135	0.015
N3	1.427	1.533	1.051	0.002	0.121	0.030
N4	8.241	8.906	8.638	0.002	0.026	0.003
N5	1.787	2.362	2.251	0.001	0.053	0.006
N6	6.331	8.010	7.801	0.001	0.101	0.011
N7	5.297	6.449	6.303	0.001	0.075	0.010
N8	8.965	11.971	11.696	0.001	0.113	0.011
RR1	2.599	4.671	4.368	0.001	0.116	0.010
RR2	1.800	2.334	2.151	0.001	0.080	0.009
RR3	4.554	4.900	6.255	0.001	0.114	0.013
RR4	1.662	3.526	3.339	0.001	0.080	0.010
RR5	0.600	3.789	4.543	0.001	0.157	0.042
RR6	1.417	2.673	3.041	0.002	0.066	0.012
RR7	0.357	2.722	2.521	0.001	0.179	0.045
RR8	1.954	1.958	2.122	0.001	0.084	0.020
RR9	1.959	2.969	2.884	0.001	0.093	0.010
RR10	3.697	7.673	5.688	0.001	0.077	0.008
RR11	1.004	2.439	1.764	0.001	0.041	0.005
RR12	0.296	3.053	2.730	0.001	0.153	0.037
RR13	0.549	2.656	7.385	0.001	0.104	0.014
RR14	2.505	4.990	2.258	0.001	0.097	0.026
RR15	0.731	8.779	8.982	0.001	0.098	0.011
RR16	0.311	1.481	9.389	0.002	0.027	0.003
RR17	0.381	1.988	2.658	0.005	0.122	0.033
RR18	0.265	5.333	2.124	0.003	0.138	0.044
RR19	0.317	2.669	4.601	0.002	0.114	0.022

RR20	0.131	2.632	8.514	0.002	0.039	0.003
RR21	0.469	6.463	10.554	0.002	0.083	0.009
RR22	0.164	3.004	7.070	0.002	0.059	0.009
RR23	1.234	5.965	10.998	0.002	0.105	0.015
RR24	1.696	9.286	1.405	0.000	0.110	0.015
RR25	1.024	9.717	8.563	0.001	0.120	0.011
RR26	0.910	10.842	9.773	0.001	0.122	0.012
RR27	0.611	7.330	10.053	0.002	0.130	0.013
RR28	0.753	11.303	7.314	0.002	0.115	0.011
RR29	0.486	8.819	8.392	0.001	0.123	0.013
RR30	0.675	10.067	4.295	0.001	0.104	0.009
RR31	0.856	10.296	1.652	0.003	0.093	0.009
RR32	0.430	7.595	5.148	0.002	0.152	0.014
RR33	0.624	8.634	6.100	0.001	0.080	0.008
RR34	0.518	4.525	5.015	0.002	0.120	0.010
RR35	0.612	6.401	12.002	0.001	0.154	0.014
RR36	0.564	5.209	9.445	0.001	0.096	0.013
RR37	0.501	12.229	7.509	0.002	0.081	0.010
RR38	1.095	9.682	6.606	0.002	0.087	0.011
RR39	0.440	7.787	2.306	0.007	0.127	0.013
RR40	0.573	6.836	2.357	0.001	0.125	0.012
RR41	1.266	9.215	8.949	0.001	0.099	0.010
RR42	0.440	6.096	5.856	0.001	0.102	0.010

Table S5: Spearman correlation results between potential biomarkers and CH_4 emissions.

**Factor**	**Spearman correlation**	***P*-value**
Acetate:Propionate ratio	0.667	0.00000
K06937	0.655	0.00000
Met Shannon Even	-0.616	0.00001
Met Shannon Div	-0.615	0.00001
K00203	0.612	0.00001
K00200	0.606	0.00001
Mitsuokella	-0.605	0.00001
K00169	0.604	0.00001
Bacteroides	-0.598	0.00001
K02117	0.598	0.00001
K00584	0.590	0.00002
Methanobrevibacter	0.582	0.00002
Acetate	0.581	0.00002
Propionate	-0.580	0.00002
K00170	0.579	0.00002
K00150	0.579	0.00002
Allisonella	-0.578	0.00003
K02118	0.576	0.00003
K00400	0.574	0.00003
Acidaminococcus	-0.573	0.00003
K00580	0.573	0.00003
Valerate	-0.571	0.00003
K01499	0.566	0.00004
K14128	0.565	0.00004
K00205	0.562	0.00005
K02303	0.561	0.00005
K13812	0.558	0.00006
Roseburia	-0.553	0.00007
K00046	0.550	0.00007
Sarcina	0.543	0.00010
K00201	0.542	0.00010
K14120	0.533	0.00014
Wenzhouxiangella	-0.531	0.00015
K00123	0.525	0.00018
Butyrivibrio	0.522	0.00020
K01959	0.522	0.00020
Singulisphaera	0.507	0.00033
Emticicia	0.504	0.00036
K00399	0.501	0.00039
Edwardsiella	-0.501	0.00040
Sulfurovum	-0.495	0.00048
Odoribacter	-0.494	0.00049
K00672	0.493	0.00050
K00581	0.490	0.00055
Methylomonas	-0.488	0.00058

Total Methanogens	0.486	0.00062
Dorea	-0.485	0.00064
Methanotorris	0.483	0.00067
Megasphaera	-0.481	0.00071
Halotalea	-0.481	0.00072
K01673	0.475	0.00085
Marinomonas	-0.474	0.00088
Endomicrobium	0.472	0.00092
Opitutus	0.468	0.00103
Colwellia	-0.465	0.00113
Dehalococcoides	-0.460	0.00130
A:B	0.449	0.00177
Salinicoccus	-0.448	0.00178
Pseudobutyrivibrio	0.448	0.00179
Veillonella	-0.444	0.00199
Alkalilimnicola	-0.434	0.00257
Isosphaera	0.432	0.00270
Olsenella	-0.431	0.00282
PCoA-2	-0.423	0.00337
Rubinisphaera	0.422	0.00346
Proteobacteria	-0.416	0.00400
Sedimenticola	-0.399	0.00605
Nitrobacter	-0.397	0.00624
Methanosphaera	0.365	0.01263
Faecalitalea	-0.364	0.01297
Euryarchaeota	0.356	0.01503
Methanobacterium	0.330	0.02516
Butyrate	0.314	0.03364
Isovalerate	-0.296	0.04574
Pelosinus	-0.281	0.05886
Zobellia	-0.278	0.06168
Methanohalophilus	-0.274	0.06502
Lactobacillus	-0.270	0.06910
Nitrosococcus	-0.264	0.07592
Kosmotoga	-0.259	0.08232
Aneurinibacillus	-0.252	0.09073
Methanocaldococcus	0.241	0.10702
Isobutyrate	-0.219	0.14278
K03389	-0.189	0.20733
K00402	-0.145	0.33629
K00401	-0.140	0.35339
Shannon diversity	0.138	0.35865
Total Methanotrophs	-0.134	0.37381
K00320	-0.133	0.37995
K14101	-0.125	0.40682
PCoA-1	0.121	0.42453
K00202	-0.116	0.44419
Methanococcus	0.107	0.47839

K00125	-0.104	0.49109
K03390	-0.100	0.50950
K07388	-0.086	0.56837
K14123	-0.084	0.57866
K00577	-0.078	0.60517
K00440	-0.065	0.66737
Shannon evenness	0.061	0.68778
Jeotgalibacillus	-0.057	0.70560
F:B	0.036	0.81445
Thalassospira	-0.035	0.81933
